# Circulating Biomarkers for the Early Diagnosis of Alzheimer’s Disease

**DOI:** 10.3390/ijms26157268

**Published:** 2025-07-27

**Authors:** Vharoon Sharma Nunkoo, Anamaria Jurcau, Mihaela Les, Alexander Cristian, Marius Militaru, Cristian Marge, Diana Carina Iovanovici, Maria Carolina Jurcau

**Affiliations:** 1 Doctoral School of Biomedical Sciences, University of Oradea, Universitatii Street nr 1, 410087 Oradea, Romania; 2 Department of Psycho-Neurosciences and Rehabilitation, Faculty of Medicine and Pharmacy, University of Oradea, 1 Decembrie Square nr 10, 410073 Oradea, Romania; 3 Department of Neuroscience, “Victor Babeș” University of Medicine and Pharmacy, Eftimie Murgu Square nr 2, 300041 Timisoara, Romania; marius.militaru@umft.ro; 4 Municipal Emergency Hospital Timisoara, 300254 Timisoara, Romania; 5 Faculty of Medicine and Pharmacy, University of Oradea, 1 Decembrie Square nr 10, 410073 Oradea, Romania; mariacarolina.jurcau@student.uoradea.ro

**Keywords:** Alzheimer’s disease, blood-based biomarkers, amyloid beta, phosphorylated tau, neurofilament light, glial fibrillary acidic protein, miRNAs, exosomes

## Abstract

With a rapidly growing incidence and prevalence, Alzheimer’s disease (AD) is rapidly becoming one of the most disabling, lethal, and expensive diseases of the century. To diagnose AD as early as possible, the scientific world struggles to find reliable and non-invasive biomarkers that could predict the conversion of mild cognitive impairment to AD and delineate the ongoing pathogenic vicious pathways to be targeted with therapy. Research supports the use of blood biomarkers, such as Aβ_1-42_/Aβ_1-40_ ratio, phosphorylated tau181, and p-tau217 for diagnostic purposes, although the cut-offs are not clearly established and can depend on the assays used. For more accurate diagnosis, markers of neurodegeneration (neurofilament light) and neuroinflammation (glial fibrillary acidic protein) could be introduced in the biomarker panel. The recent approval of the Lumipulse G p-tau217/Aβ_1-42_ plasma ratio by the FDA for the early detection of amyloid plaques associated with Alzheimer’s disease in adult patients, aged 55 years and older, exhibiting signs and symptoms of the disease represents a significant advancement in the diagnosis of Alzheimer’s disease, offering a more accessible and less invasive way to diagnose this devastating disease and allow potentially earlier access to treatment options.

## 1. Introduction

Dementia is currently the seventh cause of death and a major cause of disability and dependency worldwide [1]. Estimated global costs for dementia care in 2019 reached USD 1313.4 billion, translating in USD 23,796 per person spent for the 55.2 million people with dementia, with half of these costs being needed for informal care [2]. The outlook is even more concerning, because the prevalence of dementia is projected to double in Europe and triple worldwide by 2050 [3]. These numbers pose enormous strains on healthcare systems across the World. Generally regarded as a disease of the elderly, epidemiological studies estimate that the global age-standardized prevalence of dementia in the age range of 30 to 64 years is 119.0 per 100,000 population, which means that 3.9 million people live with young onset dementia worldwide [4].

Many forms of dementia have been identified, and their diagnostic criteria have been refined. Alzheimer’s disease (AD) is the most common form [4], although most dementia patients have mixt underlying pathologies [5,6,7,8]. Nevertheless, cardiovascular risk factors and an unhealthy lifestyle account for only 40% of the worldwide risk of any type of dementia [9].

The detailed discussion of the interlinked pathophysiological mechanisms of AD is beyond the aim of this review. Nonetheless, aging is the most prominent risk factor for late-onset AD, and many alterations in the expression and CSF or plasma levels of various molecules have been linked to aging. For example, the melatonin (a neurohormone produced by the pineal gland under influence of the hypothalamic suprachiasmatic nucleus, involved in regulating circadian rhythms) levels in the cerebrospinal fluid decreased with age in healthy persons, but this decrease was more significant in patients with AD [10]. The altered production of melatonin in AD may explain the circadian rhythm disturbances seen in AD, such as the sundowning syndrome and nightly restlessness, and may also contributed to disease progression because melatonin has anti-inflammatory, anti-oxidant, anti-fibrillogenic, anti-hyperphosphorylating, and anti-amyloidogenic properties [10]. Sirtuins, a family of enzymes performing NAD^+^-dependent protein deacetylation/diacylation, maintain brain homeostasis and prevent AD by regulating amyloid precursor protein (APP) processing, tau protein processing, mitochondrial function, the level of oxidative stress, and neuroinflammation. Their enzymatic activity and even their expression declines with age and, as such, the diminished sirtuin activity is involved in the formation of amyloid beta aggregates and neurofibrillary tangles as well as in promoting neuroinflammatory pathways [11]. As for comorbidities, hypertension is one of the most common diseases linked to cognitive decline and AD [12]. Aside from promoting hemorrhagic, ischemic, and lacunar strokes, as well as microinfarcts and microbleeds, in animal experiments, hypertension induces perivascular functional and morphological changes through which the clearance of amyloid beta and tau clearance is disrupted [13], although evidence for these changes in humans is limited and sometimes contradictory. Also in animal models, hypertension elevates the activity of beta- and gamma secretases, shifting APP processing towards the amyloidogenic pathway, and promotes tau phosphorylation by disrupting endothelial nitric oxide production and subsequent activation of cyclin-dependent kinase 5 [13]. Another commonly associated disease and a prominent risk factor for AD is diabetes mellitus (DM). Both AD and DM share insulin resistance and a reduced expression of the insulin receptor, leading some researchers to consider AD as type 3 diabetes [14]. Antidiabetic drugs, such as glucagon-like peptide 1 analogues, can significantly improve cognition in AD patients by improving insulin resistance or regulating oxidative stress and the levels of a series of inflammatory cytokines [14].

Research aimed at finding a cure for AD has been paved with many trial failures. The inability to diagnose the disease at an early stage is considered one of the main reasons for this disappointing reality [15]. Given the fact that AD pathologies begin to accumulate long time (up to 20 years) before symptom onset, current research is focusing on identifying reliable, affordable, and non-invasive markers to diagnose the disease in a preclinical stage and to develop personalized treatment strategies tailored to the various disease trajectories in each individual [16].

## 2. Diagnostic Criteria for Alzheimer’s Disease: Historical Perspective

For a long time, the diagnosis of AD could be made with certainty only postmortem, by showing the pathological hallmarks of the disease described already by Alois Alzheimer, namely, the presence of amyloid (or senile) plaques and of neurofibrillary tangles. An attempt to define clinically AD was made by the National Institute of Neurological and Communicative Disorders and Stroke and the Alzheimer’s Disease and Related Disorders Association in 1984 through identifying a series of suggestive clinical symptoms [17] (Table 1).

A combined clinical and biological diagnostic approach was proposed in 2007 by the International Working Group [18], followed by a series of revisions of the diagnostic criteria that progressively incorporated imaging and biological markers to refine and enable an earlier diagnosis of AD, as shown in Table 1 [19,20].

**Table 1:** Successively revised criteria for the diagnosis of Alzheimer’s disease.

Criteria	Applicable Setting	Clinical Presentations	Required Biological Markers	Reference
NINCDS–ADRDA (1984)	Research and clinical.	Memory changes and impairment in at least one another cognitive domain.	None.	[21]
IWG (2007)	Research.	Amnestic syndrome of a hippocampal type.	CSF biomarkers, MRI atrophy, [^18^F]-FDG-PET showing glucose hypometabolism, positive Aβ-PET, or AD autosomal dominant mutation.	[17]
IWG (2010)	Research.	Amnestic syndrome of a hippocampal type, posterior cortical variant, logopenic variant, or behavioral–frontal variant.	low CSF Aβ42, high phosphorylated tau, or high total tau, or positive amyloid PET.	[18]
NIA–AA (2011)	Research and clinical.	Mild cognitive impairment (amnestic or non-amnestic) or dementia.	Amyloid β markers (CSF or PET) or marker of degeneration (CSF tau, phosphorylated tau, [^18^F]-FDG-PET, and T1-weighted MRI).	[22]
IWG (2014)	Research.	Amnestic syndrome of a hippocampal type, posterior cortical variant, logopenic variant, or behavioral– frontal variant.	CSF amyloid β and tau or amyloid PET positive.	[23]
IWG–AA (2016)	Research.	None.	Amyloid β marker (CSF or PET) and tau marker (CSF or PET).	[24]
NIA–AA (2018)	Research.	None.	Amyloid β marker (CSF or PET) and tau marker (CSF or PET).	[25]
IWG (2021)	Research and clinical.	Amnestic variant, posterior cortical atrophy, logopenic variant primary progressive aphasia, behavioral or dysexecutive frontal variant, corticobasal syndrome, semantic and non-fluent variants of primary progressive aphasias.	Amyloid β marker (CSF or PET) and tau marker (CSF or PET).	[19]
NIA–AA (2024)	Research.	None.	Amyloid PET, CSF biomarkers and reliable plasma biomarkers (mainly p-tau217) grouped into Core 1 biomarkers, sufficient for diagnosing AD; tau-PET.	[20]

ADRDA—Alzheimer’s Disease and Related Disorders Association (now the Alzheimer’s Association) Work Group; IWG—International Working Group criteria; IWG–AA—International Working Group and Alzheimer’s Association joint criteria; NIA–AA—US National Institute on Aging and Alzheimer’s Association joint criteria; NINCDS—US National Institute of Neurological and Communicative Disorders and Stroke criteria; PET—positron emission tomography; CSF—cerebrospinal fluid; p-tau—phosphorylated tau.

Currently, the diagnostic criteria rely on the AT(N) classification system, with favored “A” (amyloid beta, Aβ) biomarkers being Aβ_1-42_/Aβ_1-40_ ratio in the cerebrospinal fluid (CSF) or plasma and Aβ-PET (positron emission tomography) imaging, “T” (tau) biomarkers being phosphorylated tau p-tau181 or p-tau217 in CSF or plasma and tau-PET imaging, and neurodegeneration biomarkers (“N”) being proposed as second-tier markers, namely, neurofilament light (Nfl), volumetric magnetic resonance imaging (MRI), and [^18^F]-fluorodeoxyglucose (FDG)-PET [21]. Tau variants phosphorylated at the mid-region (at threonines 181, 217, and 231) become abnormal usually concomitantly with the positivity of amyloid PET and before tau-PET becomes positive. The microtubule-binding region (MBTR)-tau243 becomes abnormal closer to the onset of tau-PET positivity. For these reasons, in the latest diagnostic criteria the “T” category has been split into T1, corresponding to changes in the levels of phosphorylated mid-region tau fragments, and T2, indicating changes in MBTR-tau243 and positive tau-PET. As such, biomarkers have been grouped in Core 1 and Core 2 biomarkers.

Core 1 biomarkers consist of amyloid-PET, cerebrospinal fluid (CSF) Aβ_1-42_/Aβ_1-40_ ratio, CSF phosphorylated (p)-tau181/Aβ_1-42_, CSF total tau/Aβ_1-42_, or combinations of these, and these markers are considered to be diagnostic of AD [20].

Core 2 biomarkers, consisting of changes in MTBR-tau243, p-tau205, or positive tau-PET, are not used for initial diagnosis (although they are highly associated with Aβ pathology and are considered sufficient to rule in AD), and serve mainly to stage disease severity and inform on the rate of progression [15].

Staging of the AD continuum can be achieved by PET, as shown in Table 2 [20].

Although not equivalent, some fluid biomarkers have been proposed to be used for staging the AD continuum [20]:

- For stage A, the proposed fluid biomarkers would be CSF Aβ_1-42_/Aβ_1-40_, p-tau181/Aβ_1-42_, t-tau/Aβ_1-42_, or accurate plasma assays.
- For stage B, other p-tau forms, such as p-tau205, could be used.
- In stage C, MBTR-tau243 would be altered.
- In stage D other, non-phosphorylated tau fragments could be detected in biological fluids.

Additionally, markers of inflammation (“I”), vascular damage (“V”), and α-synuclein (“S”) are proposed to be incorporated to allow for detection of comorbidities and of the multiple “molecular roads” that lead to AD [26], and for a more personalized approach in each individual patient [27]. As such, the currently used AT(N) classification system may evolve into a more complex one, incorporating markers of synaptic dysfunction and loss, oxidative stress, neuroinflammation, or vascular injury, that would be more useful in clinical trials where these comorbidities could influence the trial outcome [28].

## 3. Circulating Biomarkers for the Detection and Staging of Alzheimer’s Disease

### 3.1. Definition of a Biomarker

A biomarker can be defined as a measurable indicator (most commonly a molecule) of a biological state or condition, indicating molecular changes at both physiological and pathological levels [29]. Ideally, the biomarker should be reproducible, easy to measure, non-invasive, cost-effective, and highly accurate, able to distinguish between similar conditions [30].

Biomarkers that predict the clinical onset of AD could help identify individuals in the early, preclinical stages of the disease, allowing for more effective therapeutic interventions. Some researchers argue that the diagnosis of AD should require the presence of both abnormal biomarkers and clinical symptoms (reserving the label “at risk for AD” in the presence of only abnormal biomarkers). However, AD is not the only condition that would be diagnosed in an asymptomatic stage because it is well known that treatments are more effective when started as early as possible in the disease process. Moreover, individuals with abnormal AD biomarkers may not develop symptoms due to the increasing all-cause mortality rates with advancing age, but this should not be a reason to withhold efficient therapies despite the psychological, social, or financial discomfort that might be caused by such a diagnosis [31].

### 3.2. Plasma vs. CSF Biomarkers

The CSF has the advantage of being continuous with the cerebral extracellular space, allowing for exchange of molecules from the brain to the CSF, which is the reason why the first biomarkers for AD were fluid biomarkers. However, collecting CSF requires a lumbar puncture, which is a rather invasive method and limited to a smaller number of specialized centers.

Blood biomarkers are less invasive, more expensive, and easier to obtain even in non-specialized settings or rural areas [27]. Nonetheless, several challenges hamper the use of blood biomarkers. First, the blood–brain barrier (BBB) allows only a fraction of brain proteins to obtain access to the bloodstream, which is why the concentrations of Aβ and p-tau are much lower in the blood compared to the CSF [32]. Second, these brain-derived proteins move into a biological compartment with high concentrations of proteins that can interfere with the measurement methods [33]. Third, some of these proteins are expressed by peripheral tissues as well. For example, Aβ can be produced by the liver or originate from platelets [34]. Fourth, proteins released into the bloodstream can undergo degradation by proteases, metabolization in the liver, or can be cleared by the kidneys even before they can be measured, leading to variances not related to brain alterations [35]. Fifth, the antibodies present in the peripheral blood may react with the non-human monoclonal antibodies of the assay and generate false negative or false positive results [36]. As such, blood biomarkers were initially regarded with skepticism, due to the low sensitivity of first- and second-generation immunoassays. Nonetheless, renewed optimism has been elicited by the development of ultrasensitive platforms with increased analytical sensitivity, such as third-generation immunoassays based on electrochemiluminescence technology, or fourth-generation immunoassay such as the Single Molecule Array (SiMoA), the Proximity Extension Assay, the Immunomagnetic Reduction, or the fully automated chemiluminescence enzyme immunoassay platform [27]. Currently, the Alzheimer’s Precision Medicine Initiative (APMI) recommends the use of biomarkers to guide trials including individuals with preclinical stage disease in the context of personalized medicine [37].

### 3.3. Circulating Biomarkers for Amyloid Pathology

Amyloid beta (Aβ) results from the proteolysis of amyloid precursor protein (APP) by beta-site APP cleavage enzyme (BACE-1) followed by further processing of the resulting protein by the γ-secretase complex [38]. According to the amyloid cascade hypothesis, AD results mainly from the intra- and extra-neuronal accumulation of misfolded Aβ. The Aβ peptide contains 37–43 amino acids, with the Aβ_1-40_ being the most common isoform and the Aβ_1-42_ isoform being the most prone to aggregation and toxic one [39].

In the CSF, the content of Aβ_1-40_ normally exceeds the concentration of Aβ_1-42_ by around 10 times, and is unaltered in AD, while the concentration of Aβ_1-42_ further decreases in AD, probably because the amyloid plaques are formed mainly by Aβ_1-42_ [40]. As such, the CSF ratio of Aβ_1-42_/Aβ_1-40_ has enhanced diagnostic accuracy in differentiating AD from non-AD clinical phenotypes compared to CSF Aβ_1-42_ alone [41].

Aβ species have a significantly reduced fold change in the plasma compared to the CSF (around 10–15% versus 40–50%), due to a significant fraction of plasma Aβ originating from platelets or other non-cerebral tissues [32]. Early attempts of examining the plasma concentration of Aβ_1-42_ or the Aβ_1-42_/Aβ_1-40_ ratio in the plasma in diagnosing patients with or at risk for AD yielded inconsistent results [42], depending on the used measurement technique, pre-analytical components of the assay, storage conditions, or the number of freeze/thaw cycles [43].

The development of novel, more sensitive assays, for example, by using Aβ antibodies coated onto paramagnetic beads directed to the mid-region or N-terminal of the peptide, changed this paradigm [44]. Although the plasma Aβ_1-42_/Aβ_1-40_ ratio decreases only by 10–20% in subjects with Aβ pathology, compared to a 40–60% decrease in the CSF [45,46], evaluating this blood biomarker has been shown to have accuracy ratios between 82% and 97% when using mass spectrometry technologies, and between 62% and 79% when using immunoassays [47]. Although additional validation would be required especially in old adults with chronic comorbidities and in different racial groups, data from the Swedish BioFINDER study and from German biomarker studies showed that studying plasma Aβ_1-42_ and Aβ_1-40_ levels are able to predict Aβ pathology not only in AD, but in mild cognitive impairment (MCI) as well [48]. Adjustments for *APOE* ε4 status, age, and education increases accuracy [46]. Moreover, a soluble oligomer binding assay (SOBA) is able to detect α-sheet-containing toxic Aβ oligomers in the plasma along the different stages of early preclinical up to dementia stages of AD [49].

However, several challenges hamper the use of plasma Aβ as a surrogate measure of cerebral amyloid pathology. A large proportion of individuals had levels close to the cut-off points or within the statistical error limits (“gray zone”) [50], making the interpretation of these results difficult. Further, as already mentioned, the decrease in the plasma Aβ_1-42_/Aβ_1-40_ ratio is much less than in the CSF. Nonetheless, the use of this biomarker could significantly diminish the required Aβ-PET scans in clinical trial participant recruitment [51], especially if using an algorithm that includes *APOE* proteotype and age [52] and help clinicians in making decisions about AD diagnosis and pharmacological management [53].

Increases in the plasma Aβ_1-42_/Aβ_1-40_ have been described in both *APP* and *PSEN1* carriers compared to non-carriers. However, there are marked differences in amyloid-β ratios between genotypes: Aβ_1-42_/Aβ_1-38_ is higher in *PSEN1* versus *APP* carriers, reflecting impaired γ-secretase processivity, and Aβ_1-38_/Aβ_1-40_ is higher in *APP* versus *PSEN1* [54]. Further research is needed to establish validated cut-offs in plasma amyloid beta biomarkers in familial AD, especially as genetic variants linked to increased risk of AD are continuously identified and will hopefully help us gain more insight into the pathogenetic pathways leading to the development of AD.

### 3.4. Circulating Biomarkers for Tau Pathology

Another classical hallmark of AD are neurofibrillary tangles, composed of full-length or truncated hyperphosphorylated tau protein. With the main function of tau being the stabilization of the microtubule tracks, phosphorylation of the protein at various sites makes it prone to aggregation into paired helical filaments and destabilizes the microtubules. Neurofibrillary tangles appear first in the entorhinal cortex and spread to the limbic areas, hippocampus, and neocortex in the well-known Braak stages [55]. As a consequence of tau pathology, there is impaired synaptic transmission, axonal transport, signal transduction, which all lead to neuronal degeneration [56].

Tau hyperphosphorylated at different sites, such as threonine 181 (p-tau181), threonine 217 (p-tau217), or threonine 231 (p-tau231), can be detected in the CSF [57] of AD patients and differentiated from non-AD dementias and cognitively unimpaired individuals [47].

Plasma p-tau181 has a comparable specificity to both CSF Aβ_1-42_/Aβ_1-40_ ratio and CSF total tau and is only marginally inferior to CSF p-tau181 combined with tau-PET imaging in differentiating AD dementia from non-AD neurodegenerative diseases [58,59]. In symptomatic carriers of the *PSEN1* or *APP* mutations, plasma p-tau181 were about three times higher compared to non-carriers [60].

Plasma p-tau217 concentrations are correlated with cortical tau pathology in AD, but not in other pathologies, making this marker relatively specific for AD [61]. Moreover, plasma p-tau217 levels increase even before the abnormalities appear on tau-PET [62]. The superiority of p-tau217 compared to p-tau181 is highlighted by the larger differences in concentrations between AD patients and cognitively unimpaired individuals [63]. It has also been shown to increase in longitudinal studies only in Aβ-positive patients, with the highest increases noted in those with tau pathology [64]. As such, plasma p-tau217 was shown to be superior to plasma p-tau181 in predicting cognitive decline and monitoring disease progression in two large cohorts, the Swedish BioFINDER-1 and the Wisconsin Registry for Alzheimer Prevention (WRAP) cohorts [65]. Similar to p-tau181, plasma p-tau217 was higher in *PSEN1* mutation carriers compared to non-carriers even in the presymptomatic stage [66]. Nonetheless, sex differences were recently identified, with p-tau217 being an earlier AD biomarker in women compared to men [67]. An integrated model, combining plasma p-tau181 and p-tau217, with age, gender, APOE ε4 status, and psychological tests reliably predicted conversion from mild cognitive impairment to AD dementia in the Biomarker of AmyLoid peptide and ALZheimer’s disease Risk (BALTAZAR) study [68].

Plasma p-tau231 is a more recently examined biomarker shown to be altered earlier in the AD continuum, even before the achievement of the threshold for Aβ-PET positivity [69], but it performed inferior to p-tau217 across the AD clinical spectrum [27].

Tau aggregates composed of the microtubule binding region (MTBR) of tau are significantly associated with the clinical and cognitive symptoms of AD, and the MTBR of tau containing the residue 243 (MTBR-tau243) emerged as a novel specific AD biomarker in both CSF and plasma. CSF MTBR-tau243 had the strongest correlation with tau-PET imaging [70], as was more recently proven for plasma MBTR-tau243 too [71]. These findings make this biomarker very useful for estimating the tauopathy load in AD and for monitoring the efficacy of tau-targeted therapies in clinical trials.

Aside from the phosphorylated sites, distinction of the different truncation patterns of circulating tau fragments could capture important aspects of neurodegeneration in AD. The N-terminal tau fragment (NT1) in plasma can predict cognitive decline in elderly individuals with preserved cognitive function [72].

### 3.5. Biomarkers of Neurodegeneration

In addition to the “A” and “T” biomarkers, biomarkers for neurodegeneration (“N”) and neuroinflammation (“I”) have been proposed as second-tier markers due to their non-specific nature, because they are present in a large array of neurodegenerative diseases [27]. To date, validated “N” biomarkers include neurofilament light (Nfl), volumetric MRI, and [^18^F]-FDG-PET, although other markers are subject to active research [27].

NfL belongs to the neuronal cytoskeletal proteins, together with neurofilament medium, neurofilament heavy chains, α-internexin, or peripherin [73]. NfL assembles into neurofilaments that are important for stability and growth of axons as well as for dendritic branching [37]. Among all these neurofilaments, NfL spreads more easily from the brain parenchyma into the CSF in case of axonal degeneration due to its smaller molecular weight and increased solubility. The severity of neurodegeneration correlates with elevated NfL concentrations in the CSF [74] as well as in the blood [75,76]. Moreover, the diagnostic performance is similar for measurements of NfL both in the blood and CSF [77].

Plasma NfL levels increase even in the preclinical stage and rise more consistently when the onset of cognitive decline approaches [78]. With the progression of AD, there is an increase in the plasma concentrations of NfL, correlating with amyloid and tau biomarkers, MRI measurements (regarding both white and gray matter loss), as well as with cognitive performances [79]. The usefulness of NfL in evaluating and monitoring AD has been confirmed in studies of both late-onset, sporadic AD [80,81] and familial AD [82].

Nonetheless, it should be kept in mind that NfL is a non-AD-specific marker of neurodegeneration, increasing in non-AD forms of dementia as well [83]. Moreover, the plasma levels of NfL have been shown to increase with age [84], depend on gender (higher in female carriers of PSEN1 mutations compared to male carriers) [85], and vary with comorbidities such as kidney failure or diabetes. As such, the establishment of age-dependent cut-off points [86] would be advisable.

### 3.6. Biomarkers of Neuroinflammation

#### 3.6.1. Glial Fibrillary Acidic Protein (GFAP)

Glial fibrillary acidic protein is a filamentous protein serving as a cytoskeletal constituent of astrocytes of the central nervous system (CNS) and in non-myelinating Schwann cells of the peripheral nervous system. Because neuroinflammation has been increasingly implicated in the pathogenesis of aging [87] and neurodegenerative diseases, including AD [88], it is not surprising that researchers have turned to evaluating neuroinflammation as a marker of AD progression. Indeed, the concentrations of GFAP are increased in both CSF and plasma of AD patients [89]. Moreover, plasma GFAP can more accurately distinguish Aβ-positive from Aβ-negative individuals compared to CSF GFAP [90] and increases even before plasma p-tau and NfL in sporadic [91] and familial forms of AD [92].

A series of studies have shown that GFAP predicts conversion to AD in MCI individuals [93] and that tau accumulation occurs only in cognitively normal Aβ-positive individuals with increased levels of GFAP [94]. In fact, GFAP is a marker of astrocytic activation, which can be triggered by Aβ aggregates accumulating in the brain, and GFAP concentrations increase linearly with increasing Aβ-PET burden [95].

Although neuroinflammation is a universal accompaniment of neurodegeneration, GFAP has been shown to reliably differentiate AD from behavioral variant frontotemporal dementia (bvFTD) with a specificity of 79% and a sensitivity of 89% [96] or from FTD and progressive supranuclear palsy with an area under the curve (AUC) of 0.84 and 0.81, respectively [97].

All these findings point to plasma GFAP as a possible specific biomarker for Aβ pathology, allowing for discrimination between AD and non-AD clinical phenotypes, as well as for early diagnosis of AD [90].

#### 3.6.2. Triggering Receptor Expressed on Myeloid Cells 2 (TREM2)

TREM2 is a receptor expressed on the surface of microglial cells which can produce soluble TREM2 (sTREM2) following shedding of its ectodomain [38]. Upon microglial activation, the expression of TREM2 is reduced and sTREM2 is excreted into the CSF [27]. Although most studies have shown increased amounts of sTREM2 in the CSF [98], recent studies showed increased TREM2 mRNA levels in the peripheral mononuclear cells dependent on the *APOE* genotype [99]. TREM2 can be measured in the plasma, allowing for a diagnostic accuracy of 70% for AD [100]. The soluble ectodomain can also be evaluated in bodily fluids; accumulation of Aβ and increased CSF p-tau levels were associated with low levels of plasma sTREM2, but a similar trend also characterized vascular dementia [101]. Leukocytes also express TREM1, and the plasma levels of sTREM1 behave differently, rising in AD and correlating with AD severity [102]. As such, more research is needed to clarify the role of TREM as blood biomarker in AD [30].

#### 3.6.3. Chitinase-3 Like-Protein 1 (CHI3L1)

CHI3L1, also known as YKL-40, is a pro-inflammatory glycoprotein expressed in glial cells and secreted by astrocytes in response to stress signals or neuroinflammatory stimuli [103]. Its CSF and plasma levels are increased across the AD continuum, correlating with tau-PET burden but not with Aβ pathology [104], and have been shown to discriminate between normal and mildly impaired cognitive individuals with 85% sensitivity and specificity [105], although it proved less useful for monitoring disease progression. In addition, plasma levels of CHI3L1 have been found elevated in a series of other dementias, as well as in aging [83,103].

#### 3.6.4. Monocyte Chemoattractant Protein-1 (MCP-1)

MCP-1, also known as CCL2, is a glial-derived pro-inflammatory chemokine that attracts microglia and peripheral leukocytes to the sites of inflammation. While CSF MCP-1 is considered to be a microglial biomarker, blood MCP-1 reflects activation of peripheral monocytes/macrophages and has been shown to associate faster cognitive decline in AD [106].

#### 3.6.5. Other Inflammatory Biomarkers

A series of other inflammatory cytokines have been shown to be dysregulated in the cerebral tissues and CSF of MCI and AD patients, boosting the interest for their study in the blood. Despite a sizeable body of evidence on the differences of blood cytokine levels in AD, MCI, and non-demented patients, the conclusions are still a subject of debate [30]. Studies performed so far describe elevated levels of pro-inflammatory cytokines such as interleukin IL-6, IL-1β, Il-12, IL-18, transforming growth factor (TGF)-β, tumor necrosis factor (TNF)-α [107] or IL-2, CXCL10, C reactive protein, and interferon-γ [108] in the blood of AD patients, while other studies show no significant differences between AD and non-AD subjects in the peripheral levels of IL-1β, TNF-α, IL-6, or C reactive protein [109]. Further, IL-6, IL-8, IL-10, GFAP, and TNF-α were shown to positively correlate with the Aβ_42_/Aβ_40_ ratio, p-tau181, and mainly with NfL [110]. These findings may appear confusing, as IL-8 is a well-known pro-inflammatory cytokine contributing to phagocytic cell recruitment, but Il-10 is an anti-inflammatory cytokine, suppressing T cell recruitment and pro-inflammatory interferon γ signaling. High levels of IL-2 were shown in one study to predict a slower cognitive decline in MCI patients [111].

In reviewing these conflicting results, one must acknowledge that it is difficult to separate variations of these cytokines and chemokines due to AD from those caused by comorbidities and age, which could likely contribute to the increases of these cytokines. However, in mouse studies, increases in the levels of IL-10 worsened Aβ deposition and cognitive functions [112]. In our opinion, it is unlikely that these markers would enter blood diagnostic panels for AD soon.

Interesting findings come also from the study of the gut–brain axis and of the gut microbiota. An abundance of *Allisonella*, *Lachnospiraceae FC020* group, and *Sellimonas* genera was shown to increase the risk of AD, with *Allisonella* being associated with an increase in systemic markers of inflammation [113].

### 3.7. Novel Blood Biomarkers

The omics sciences effectively explore the molecular modifications that occur in multiple systems, from subcellular compartments to large biological networks. High-throughput omics/multi-omics platforms have been developed to validate multi-variate signatures of peripheral blood candidate biomarkers and dissect the altered molecular and cellular pathways of AD [27]. This would allow the stratification of patients into biologically homogenous subsets along the AD continuum and the introduction of pathway-based, specific treatments for each stage [114].

For example, a study performed on a Chinese cohort in Hong Kong used a high-throughput proximity extension assay and classified the AD-associated plasma proteins into 19 protein clusters, each indicating a specific pathophysiological pathway. Establishing a blood-based biomarker signature from the whole plasma proteomic profile of AD allowed for accurate discrimination between AD and healthy controls [115]. A more recent study used mass-spectrometry-based proteomics and identified 138 proteins that showed different concentrations in AD compared to healthy controls. A series of biomarkers were useful for diagnosis (MBP, BGLAP, APoD), while others could be used to delineate disease progression (CLNS1A, CRISPLD2, GOLPH3) [116]. Given the multitude of these proteins and large amount of data generated by these analyses, the multiple protein clusters would need complicated algorithms to be separated, a task that could be achieved by using machine learning. However, it is unlikely that these biomarkers would enter clinical practice soon.

Multiple fatty acids (stearic, palmitic, oleic, and linoleic acids) have also been found to differ between healthy controls and patients with physio-cognitive decline (including also physical frailty) in the absence of differences in fat percentage, body mass index, or levels of HDL-cholesterol, LDL-cholesterol, or triglycerides [117]. A potential role for mitochondrial dysfunction in the pathogenesis of this syndrome may be indicated by the elevated levels of palmitoyl carnitine, a molecule involved in the transport of fatty acids for mitochondrial β-oxidation.

### 3.8. MicroRNAs as Potential Biomarkers for AD

MicroRNAs are non-coding RNAs, composed of 18–25 nucleotides, that post-transcriptionally regulate the expression of genes. Their generation starts with RNA polymerase II, which transcribes a long polyadenylated and capped RNA, known as primary miRNA (pri-miRNA), that is recognized in the nucleus by Drosha and DGCR8 and cleaved by this complex into hairpin RNAs, known as precursor miRNAs (pre-miRNAs). Pre-miRNAs are exported from the nucleus by exportin 5 and processed in the cytoplasm by Dicer (RNase III) to form 18–25 nucleotide-containing double-strand RNAs, which are further loaded into the RNA-induced silencing complex RISC, where one strand is discarded and the other guides RISC to the mRNA target [118].

Micro-RNAs can act as epigenetic modulators by targeting enzymes that can modulate gene expression such as DNA methyltransferases, histone deacetylases, or histone methyltransferases. Each miRNA can target hundreds of messenger RNAs (mRNAs) [119]. Conversely, epigenetic modifications (DNA methylation, as occurs in aging, histone modification, or RNA modifications) can directly regulate miRNAs [87,119].

By regulating microtubule-associated tau protein (MAPT) expression, tau splicing, post-translational modifications of tau, and APP expression and processing, as well as inflammation, miRNAs are likely significantly involved in AD pathogenesis and emerge as potential biomarker candidates [120]. Research has shown miR-23a and miR-125b to be increased in the CSF and serum of AD patients, being able to differentiate AD from frontotemporal dementia [121]. Altered levels in both CSF and plasma were observed for miR-34a and miR-34c as well, being able to distinguish AD cases with 83% accuracy [122]. In longitudinal studies, miR-206 was found able to predict the conversion of MCI to AD within 5 years [123], while miR-181a and miR-146a levels could identify between patients with stable MCI and those who will progress to AD within 2 years [124]. In a more recent study, 17 plasma miRNAs were found to be differentially expressed in association with CSF biomarkers of amyloid load (“A”), tau pathology (“T”), and neurodegeneration (“N”) [125]. Table 3 shows these correlations, found by Liu and coworkers [125].

However, rather than focusing on individual miRNAs, a more useful approach is to identify unique miRNA signatures that could indicate multiple deregulated pathways leading to the clinically overt disease. By analyzing the Alzheimer’s Disease Neuroimaging Initiative (ADNI) cohort, Krüger and coworkers [126] were able to sequence miRNAs and identify a series of specific signatures:

- miR-142.3p, miR-98.5p, and miR-9985 yielded an area under the curve (AUC) of 0.72 for AD.
- miR-590.3p, miR-369.3p, and miR-9985 predicted early mild cognitive impairment with an AUC of 0.71.
- miR-1306, miR-4429, and miR-22.5p characterized late mild cognitive impairment with an AUC of 0.71.

Conversion of early MCI to AD within 2 years was predicted by a cluster of miR-125.5b, miR-18a.5p, and miR26b.5p with an AUC of 0.70 (more sensitive compared to CSF biomarkers, who had an AUC of 0.60–0.62), while conversion of late MCI to AD was characterized by a signature combining expression levels of miR-338.30, miR-584.5p, and miR-142.3p with an AUC of 0.75 [126].

MicroRNA 142.3p, shared by the signatures of AD and late MCI-AD converters, is believed to play a role in neuron–microglia crosstalk and is involved in downregulation of brain-derived neurotrophic factor in activated microglia [127]. MiR-125.5b, one of the miRNAs identifying patients with early MCI at risk for conversion to AD, regulates synaptic plasticity, and its overexpression is associated with tau hyperphosphorylation and memory deficits [128]. Another miRNA of the cluster predicting conversion of early MCI to AD, miR-26b.5p, acts via cyclin-dependent kinase 5 upregulation, tau phosphorylation, aberrant cell cycle entry, and neuronal death [129], although its role has been discussed in cancer as well as in cerebral ischemia-reperfusion injuries [130,131].

### 3.9. Studies of Circulating Extracellular Vesicles

As discussed above, currently the diagnosis of AD relies on costly and invasive CSF analyses and PET scans. Researchers are trying to identify reliable biomarkers to be identified and quantified in the blood or plasma of patients, but low concentrations and the possibility of other sources of the biomarker molecules cannot be ignored.

Exosomes are extracellular vesicles (EVs) released by all types of cells that play a critical role in intercellular communication [132]. Neuronal-derived extracellular vesicles (NDEVs) contain proteins, nucleic acids, miRNAs, and other cellular components that are involved in various physiological or pathological processes such as dissemination of pathological molecules, neuroinflammation, or synaptic dysfunction [133]. Being nanoscaled particles surrounded by a lipid bilayer [134], they can cross the blood–brain barrier [135], be harvested from the blood, and analyzed to obtain a molecular snapshot of the brain [136]. However, the collection of neuron- and astrocyte-derived EVs is expensive, which is why identifying biomarkers in bulk exosomes could provide a more convenient and low-cost approach [137].

The technical procedures for collecting exosomes are well described in the literature [138] and a series of commercial kits for isolation and enrichment of exosomes are available. Isolating EVs that contain neuron-specific surface proteins allows for analysis of only neuron-derived exosomes. One example of such a protein commonly used for selecting NDEVs is L1 cell adhesion molecule (L1CAM, or CD171), a surface marker expressed mainly by neurons. Unfortunately, L1CAM is also expressed by kidney cells, or peripheral lymphocytes, raising concerns regarding the origin of the EVs [139]. More specific markers are growth-associated protein 43 (GAP43), an axonal marker, and neuroligin3, a neuron cell surface marker [139].

Analyzing the proteome and miRNAs contained in isolated and enriched exosomes can offer interesting information regarding the ongoing pathogenic cascades at a specific moment. The abnormal cleavage of APP by BACE-1 occurs in the endosomes, and the protein fragments bind to exosomes and are secreted into the extracellular space to form amyloid depositions. The accumulation and secretion of tau also involves exosomes. As such, exosomes absorb more Aβ and tau in AD patients’ brains and release them into the peripheral blood, where these proteins can be found increased in NDEVs even 10 years before symptom onset [140]. Comparing healthy controls and patients diagnosed with AD according to the NIA-AA criteria, NDEVs of AD patients had other upregulated proteins as well, involved in control of synaptic density and synaptic pruning (complement components), components of the membrane attack complex (shown to co-localize with Aβ plaques and tau tangles), proteins that act as receptors for von Willebrand factor and were associated with endothelial dysfunction (such as platelet glycoprotein Ib beta chain), and upregulated levels of Ras suppressor protein 1 (RSU1) [137]. The latter (RSU1) has been demonstrated to inhibit the c-Jun N-terminal kinase (JNK) pathway, linked to APP processing, and to enhance the extracellular signal-regulated kinase (ERK) pathway, involved in neuroprotection against Aβ toxicity and oxidative stress [141]. As such, increased RSU1 levels may reflect a compensatory response to the excess Aβ accumulation. Downregulated proteins consisted of α-2-macroglobulin, A disintegrin and metalloproteinase domain 10 (ADAM10), α-1-acid glycoprotein 2, immunoglobulin heavy chains [137], syntaxin-1, GluR2 (glutamine receptor subunit 2), PSD95 (postsynaptic density 95), and pro-BDNF (brain-derived neurotrophic factor) [139]. ADAM10 is the major α-secretase for APP processing and has been shown to play a role in preserving synaptic function and to promote hippocampal neurogenesis [142,143]. A meta-analysis of studies comparing the content of exosomes derived from patients with AD, MCI, and healthy controls revealed additional differences. Complement effector proteins are increased, while complement regulatory proteins (CR1, CD59, CD46) were decreased in the NDEVs of patients with AD [144]. Additionally, molecules related to disordered metabolism (P-S321-IRS-1, or insulin receptor substrate-1 phosphorylated at serine 312), autophagy-lysosomal dysfunction (cathepsin D, lysosome-associated membrane protein 1), vascular injury (VEGF-D, vascular endothelial growth factor D), deficiency of neurotrophic factors and neural growth (fibroblast growth factors 13, 14, and type 1 insulin-like growth factor-1) were increased, and molecules related to synaptic dysfunction, such as neurogranin, growth-associated protein 43 (GAP43), and synaptosomal-associated protein-25 (SNAP25) were significantly lower in EV samples collected from AD patients and could be valuable biomarkers for diagnosis as well as for monitoring disease progression [144,145].

EVs also contain miRNAs that can yield diagnostic and prognostic information. Addressing this issue, Visconte and coworkers [146] reported significantly increased expression levels of miR-106a-5p, miR-16-5p, miR-17-5p, miR-195-5p, miR-19b-3p, miR-20a-5p, miR-223-3p, miR-25-3p, miR-296-5p, miR-30b-5p, miR-532-3p, miR-92a-3p, and miR-451a in AD patients compared to healthy controls. Moreover, significant correlations were identified between Aβ42 and miR-30b-5p, and between tau and miR-223-3p. Analysis of miRNAs allow us also to differentiate between cognitive impairment due to different pathologies. While miR-16-5p, miR-25-3p, miR-92a-3p, and miR-451a were increased in EVs of MCI due to prodromal AD, these were not increased if the MCI was caused by non-AD pathologies [146].

Looking at the targets of these deregulated miRNAs helps us understand in more depth the temporal profile of AD pathogenesis. Gene ontology analysis revealed many of these deregulated miRNAs to be related to G-protein coupled receptor signaling pathway and protein phosphorylation, which could explain the deposition of Aβ [147], abnormal phosphorylation of tau protein [148] through various downstream kinases, such as glycogen synthase kinase 3beta (GSK-3β) or the ERK signaling cascade [149]. Other pathways regulated by the differently expressed miRNAs were apoptosis, synaptic function of glutamatergic, cholinergic, and GABA-ergic synapses and long-term potentiation, as well as TNF- and IL-17 signaling, or cytokine–cytokine receptor interaction [149], highlighting the involvement of neuroinflammation in the pathogenesis of AD [88].

## 4. Critical Appraisal of the Use of Novel Biomarkers for Diagnosing Alzheimer’s Disease

Given the rising incidence of neurodegenerative diseases, AD included, and the improved response to therapy started as early as possible, as well as the design of novel and more effective therapies for AD, the medical and scientific community struggles to identify new, more accessible, and cost-effective biomarkers that would enable diagnosis in a preclinical phase. The advantages and limitations of these markers are shown in Table 4.

In addition, saliva and tear biomarkers have also been proposed, being collected even less invasively than blood, but the collection procedures have not been standardized, and the concentration of the evaluated biomarkers is subject to variations with circadian rhythms, environmental factors, medication, or systemic comorbidities [150].

### 4.1. Selecting Between Available Laboratory Methods

Various analytical platforms have been used to measure plasma AD biomarkers since the 1990s. The early enzyme-linked immunosorbent assays (ELISA) yielded inconsistent results, failing to show correlations between CSF and plasma Aβ concentrations [151]. In 2010, a single molecule enzyme-linked immunosorbent assay using arrays of femtoliter-sized reaction chambers, known as Simoa, was developed by Quanterix Billerica, MA, USA [152], and shown to detect proteins at much lower concentrations. Alternatively, the Multiple Analyte Profiling technology (xMAP) allowed for concomitant measurements of multiple biomarkers (Aβ_1-40_, Aβ_1-42_, total tau, as well as p-tau isoforms) [153], but, again, the correlations between plasma and CSF levels were non-significant [154].

Currently, fully automated electrochemiluminescence (ECL)-based assays are increasingly replacing the more traditional ELISA and xMAP technologies, showing comparable performances with the Simoa assay for detection of Aβ and p-tau at lower costs [155].

**Figure 1:**
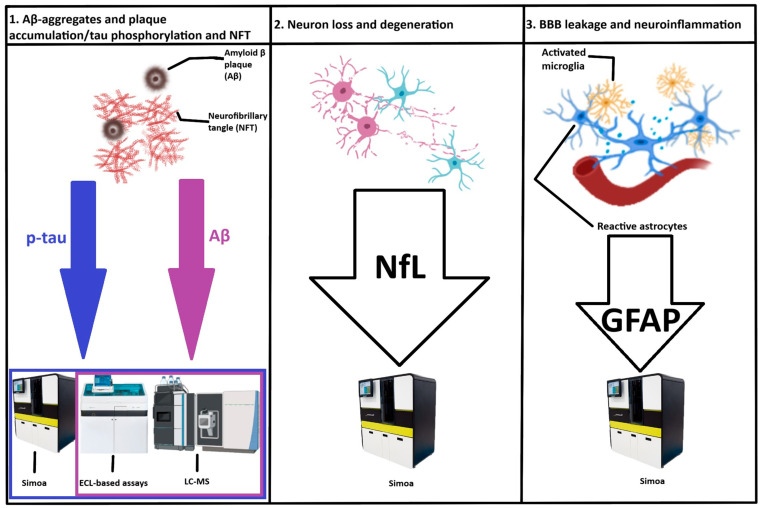
Summary of the analytical platforms with best performances in detecting and measuring blood-based biomarkers in the diagnosis of Alzheimer’s disease. BBB—blood–brain barrier; LC-MS—liquid chromatography-mass spectrometry; ECL—electrochemiluminescence; Simoa—single-molecule array; p-tau—plasma phosphorylated tau; Aβ—amyloid beta; GFAP—glial fibrillary acidic protein; NfL—neurofilament light.

Immunoprecipitation and mass spectrometry (IP-MS) tests can reliably quantify proteins, and interfacing such MS-based methods with liquid chromatography (LC-MS) adds to the accuracy of the technology (Figure 1) [156].

Nonetheless, these highly sensitive platforms are still expensive and have limited availability, which currently restrict their use in clinical settings, but show the promise of becoming valuable tools used for clinical trial inclusion, monitoring treatment effects, or even screening elderly patients with MCI for the risk of developing AD.

### 4.2. Establishing Validated Cut-Offs for the Selected Biomarkers

#### 4.2.1. Plasma Amyloid-β

Several studies evaluated the plasma levels of Aβ_1-40_, Aβ_1-42_, and Aβ_1-42_/Aβ_1-40_ ratio in AD with different platforms, showing consistently altered levels with gradually increasing accuracy [157]. Among the different assays, the LC-MS method showed the best diagnostic performance [158]. However, the cut-offs used varied quite widely, as shown in Table 5, and cut-offs for these parameters in familial cases of AD should also be established.

#### 4.2.2. Plasma Phosphorylated Tau

Many individuals can have amyloid pathology in the brain without clinical signs of cognitive impairment. In fact, tau pathology burden correlated better with cognitive decline compared with amyloid burden [163]. However, total tau is not specific for AD, increasing in a series of other conditions collectively known as tauopathies. Measuring the levels of phosphorylated tau at specific sites (p-tau181, p-tau217, p-tau231, as outlined in the previous section) is more specific for AD [164]. All three isoforms of p-tau were significantly higher in AD patients than in cognitively unimpaired individuals and were superior to plasma Aβ biomarkers in this task [165], but p-tau217 was the most sensitive marker to predict conversion of MCI to Alzheimer’s dementia [166]. The cut-offs and thresholds used by the various studies evaluating the diagnostic value of the p-tau isoforms are shown in Table 6. However, these studies were performed mainly in patients with sporadic AD, with cut-offs for familial forms of AD being further needed to find reliable cut-offs in these patients as well.

#### 4.2.3. Other Promising Biomarkers

Adding *APOE* genotype and measuring plasma NfL levels can add to the sensitivity and specificity of the above-discussed biomarkers. However, the *APOE* status alone is insufficient to act as a biomarker for the diagnosis of AD and is applicable only in combination with other biomarkers [173]. Ultrasensitive immunological assays and MS-based methods can quantify the plasma concentrations of NfL and offer important information on the neurodegenerative processes occurring in the patient’s brain, which, combined with more specific biomarkers for AD, can help in diagnosing even preclinical cases. Gerards and coworkers used a Simoa-based assay and reported an 80% sensitivity and 67% specificity for plasma Nfl levels ≥ 12.7 pg/mL [172]. Palmqvist and coworkers, working on three distinct cohorts (an Arizona-based neuropathology cohort (cohort 1), including 34 participants with AD and 47 without AD, the Swedish BioFINDER-2 cohort (cohort 2), including cognitively unimpaired participants (n = 301) and clinically diagnosed patients with mild cognitive impairment (MCI) (n = 178), AD dementia (n = 121), and other neurodegenerative diseases (n = 99), and a Colombian autosomal-dominant AD kindred (cohort 3), including 365 *PSEN1* E280A mutation carriers and 257 mutation noncarriers) and the same Simoa assay reported that for the first cohort a cut-off value of 41.9 pg/mL had a sensitivity of 82% and a specificity of 32%, while in the second cohort a cut-off of 26.5 pg/mL for NfL had a sensitivity of 67% and a specificity of 38% [174]. These findings highlight the difficulties in finding a cut-off value applicable for the differentiation between subjective cognitive decline and AD across the AD continuum.

Glial fibrillary acidic protein (GFAP) as a marker of glial activation was first reported to increase and to correlate with cognitive impairment in AD patients in 2019 [96] and has since been validated as a valuable biomarker for AD, although with significant variability. The plasma levels of GFAP correlate better than CSF levels with brain amyloid pathology [175] and with faster cognitive decline [176]. Adding GFAP consistently improves the sensitivity and specificity in differentiating AD from cognitively unimpaired subjects and from MCI [177].

### 4.3. Issues to Be Addressed Before Proceeding to Clinical Implementation of Biomarkers for Alzheimer’s Disease Diagnosis and Monitoring of Progression

From the above-presented findings, one may ask himself which analytical platform performs best in quantifying plasma biomarkers for AD. Being a complex biological compound, plasma biomarker study may come with a series of challenges related to the presence of comorbidities and aging, the metabolization and elimination dynamics of various proteins, the presence of blood proteases, or the handling of samples [155,178]. For example, chronic kidney disease significantly interferes with the plasma p-tau and NfL levels [179]. Moreover, significant inter- and intra-assay differences preclude the establishment of a unified cut-off for these biomarkers, although a combination of biomarkers in a panel can limit this variability with the downside of adding to the cost of the panel [180,181].

Second, given the many techniques available for analyzing plasma biomarkers, the question of which would be the best available platform arises. Head-to-head comparisons seem to indicate that mass-spectrometry-based assays perform better than immunoassays in measuring both Aβ_1-42_/Aβ_1-40_ ratio and p-tau levels, though they are more expensive [156,158]. Nonetheless, lower costs would facilitate widespread implementation.

Regarding the cut-off values accepted for the various biomarkers, they depend on the analytical method used and the reference cohort. Further studies could validate these values in different populations considering also ethnicity and gender, as well as comorbidities, age, or other specific differences [182]. For example, caloric restriction, shown also to be a useful rejuvenation method by promoting autophagy [183], has been shown to decrease the circulating pro-inflammatory biomarkers [184] and could interfere with the possible future use of “I” markers.

When using biomarkers, it is not uncommon for clinicians to be confronted with mismatches (“gray zones”) and to find dubious results. On the other hand, when judgement is based only on clinical criteria, studies have shown high rates of misdiagnosis [185], and many clinically diagnosed AD patients fail to show the characteristic neuropathological findings at postmortem assessments [186]. As such, current recommendations advise to use clinical assessment in combination with biomarkers and to longitudinally monitor the changes of the biomarkers [166].

Finally, there is no infallible biomarker, although plasma p-tau biomarkers, and especially p-tau217 and p-tau231, appear to show the strongest correlation with Aβ pathology [187]. Rather, a combination of biomarkers can improve screening in clinical practice. For example, combining plasma Aβ_1-42_/Aβ_1-40_ ratio with plasma p-tau181 and the *APOE* status has been shown to predict progression to AD dementia in 6 years with and AUC around 0.9 [188].

## 5. Future Directions

The identification of plasma biomarkers for AD was a breakthrough in the field, and research in this area is advancing at high speed. Although studies still need to investigate sex differences [189] and focus also on minority racial groups to establish the predictive ability of biomarker algorithms in these groups as well [190], it could allow access to more potent therapies more early in the disease course even in developing countries, which cannot afford the costly diagnostic procedures currently used. For example, an Aβ-PET currently has a cost of USD 3000–8000, for a CSF measurement of Aβ and p-tau assay one spends between USD 200 and USD 1000, while a blood Aβ MS assay would cost around USD 100–500 [155,191]. In addition, blood tests would be less invasive, and the samples could be frozen and stored in specific conditions and further processed in specialized laboratories. Using blood biomarkers for including patients in randomized clinical studies would also reduce the costs and time for enrollment [192]. When comparing amyloid PET with plasma biomarkers such as Aβ_1-42_/Aβ_1-40_, p-tau217, and particularly the p-tau217/Aβ_1-42_ ratio, they appear to have strong potential as amyloid PET alternatives and may detect amyloid accumulation even earlier than Aβ PET visual reading threshold [193].

With three anti-Aβ monoclonal antibodies (Aducanumab, Lecanemab, and Donanemab) approved for the treatment of AD and other antibodies in research (although anti-tau antibodies failed), it is imperative to start treatment as soon as possible, before the full development of the multiple interlinked pathogenic cascades, for optimal therapeutic results [194]. Moreover, longitudinal studies on a Swedish cohort revealed that characteristic alterations in the plasma biomarkers of AD are also associated with a progressive decline in muscle strength, allowing for preventive treatment of sarcopenia [195].

Research continues to identify novel biomarkers. Different genetic loci, such as the coat protein complex I G2 (COPG2), or the WW domain-containing oxidoreductase (WWOX) genes can be combined with already well-established biomarkers to increase the efficacy of predictive models for the diagnosis of AD [196].

In view of the rapid expansion of research on blood-based biomarkers for AD, it is foreseeable that these will be integrated in the diagnostic tests in specialized memory clinics in the next 3–5 years, making diagnosis possible not only in tertiary or academic centers. The recent approval of the Lumipulse G pTau217/ß-Amyloid 1–42 plasma ratio for the early detection of amyloid plaques associated with Alzheimer’s disease in adult patients, aged 55 years and older, exhibiting signs and symptoms of the disease may be only a first step in this direction [197]. Further, in (hopefully) 5–10 years, these testing panels could be used as screening tests in primary care settings, selecting those patients who would need referrals to memory clinics. As for widespread population screening, it is unlikely that these tests would be available within the next 10 years because they would need further development to reach a near-100% accuracy before low cost and disease-modifying treatments should be widely available [47].

## 6. Conclusions

With the further development of fully automated immunoassays or mass-spectrometry-based assays, the measurements of various blood-based biomarkers will become more affordable, and their sensitivity and specificity will increase.

Given the fact that more and more genetic variants that increase susceptibility to various diseases are described, the scientific community has started debating whether whole genome sequencing (WGS) could be extended beyond diagnosing rare diseases or the identification of actionable cancer drivers. The development of next-generation sequencing has enabled the analysis of entire genomes in a fast and cost-effective manner. Despite the ethical concerns raised, the data could be archived safely and reanalyzed and reinterpreted several times during the patient’s life for different clinical purposes, with the prevention of AD included. Using machine learning and artificial intelligence, polygenic risk scores could be developed [198], allowing us to select patients at high risk who could be screened with blood-based biomarkers for AD earlier, while for the rest of the patients, screening with blood-based biomarkers could be performed by the primary care physician at older ages or when a patient complains of subjective cognitive decline. The UK government’s Newborn Genomes Programme, aiming to sequence the genomes of 100,000 newborns, might be just the first step in this direction [199].

Finally, by using these various screening methods, disease-modifying treatments could be started early in the Alzheimer’s disease continuum, hoping for better therapeutic results and improved quality of life for both patients and family members.

## Figures and Tables

**Table 2:** Biological staging of AD by PET [20].

Stage	Amyloid-PET	Tau-PET Medial Temporal	Tau-PET Moderate Neocortical Uptake	Tau-PET High Neocortical Uptake	AT2 Notation
**A**	+	−	−	−	A + T2−
**B**	+	+	−	−	A + T2_MTL_+
**C**	+	+	+	−	A + T2_MOD_+
**D**	+	+	+	+	A + T2_HIGH_+

Stage A (initial)—abnormal Aβ-PET with no uptake on tau-PET (A + T2−); Stage B (early) abnormal Aβ-PET with tau-PET uptake restricted to the medial temporal areas (A + T2_MTL_+); Stage C (intermediate)—abnormal Aβ-PET with moderate tau-PET uptake in a neocortical region of interest (A + T2_MOD_+); Stage D (advanced)—abnormal Aβ-PET with high tau-PET uptake in the same region of interest (A + T2_HIGH_+).

**Table 3:** Correlations of miRNAs with amyloid, tau pathology, and markers of neurodegeneration.

Pathology	miRNA	Modification	Significance
Amyloid burden	hsa-miR-145-5p	increased	*p* < 0.001
	hsa-miR-483-3p	increased	*p* < 0.001
	hsa-miR-374a-3p	increased	*p* < 0.001
	hsa-miR-339-5p	decreased	*p* < 0.001
	hsa-miR-652-3p	decreased	*p* < 0.001
	hsa-miR-95-30	decreased	*p* < 0.001
	hsa-miR-628-5p	decreased	*p* < 0.001
	hsa-miR190a-5p	decreased	*p* < 0.001
	hsa-miR-3679-5p	decreased	*p* < 0.01
Tau pathology	hsa-miR-1224-5p	increased	*p* < 0.001
	hsa-miR-337-5p	increased	*p* < 0.001
	hsa-miR-1180-3p	increased	*p* < 0.001
	hsa-miR-190a-5p	increased	*p* < 0.001
	hsa-miR-1255b-5p	decreased	*p* < 0.001
	hsa-miR-369-5p	decreased	*p* < 0.01
Neurodegeneration	hsa-miR-1224-5p	increased	*p* < 0.001
	hsa-miR-337-5p	increased	*p* < 0.001
	hsa-miR-1180-3p	increased	*p* < 0.001
	hsa-miR-1255b-5p	decreased	*p* < 0.001
	hsa-miR-941	decreased	*p* < 0.001
	hsa-miR-369-5p	decreased	*p* < 0.001
	hsa-miR-215-5p	decreased	*p* < 0.001

**Table 4:** Advantages and limitations of currently used or proposed biomarkers for Alzheimer’s disease (adapted from Kaštelan et al. [150]).

Biomarker	Advantages	Limitations
Genetic tests	Risk assessment. Insight into an individual’s genetic Predisposition.	Cost and accessibility. Incomplete penetrance and gene expression. Interactions with other risk factors. Limited predictive accuracy.
Brain MRI	Detailed information on microstructural and functional alterations of the brain. Quantification of brain atrophy Monitoring of disease progression.	Expensive and has limited availability. Expertise required for analysis of images. Requires patient cooperation, time-consuming. Side effects of contrast material.
PET scans	Objective assessment of extent and distribution of Aβ and tau pathology in the brain. Monitoring disease progression. Differentiation of AD from other dementias.	Expensive and with limited availability. Exposure to ionizing radiations. Expertise required for the analysis of images. Time-consuming. Contrast side effects.
CSF bio- markers	High concentration of biomarkers due to proximity to the brain. Standardized procedures. Can test numerous biomarkers. Allows for longitudinal monitoring of disease progression. Can be used as outcome measure to assess efficacy of therapies.	Invasive procedure, requiring hospitalization. Risk of complications. Biomarkers may vary with age, gender, underlying health conditions, making the interpretation of the results rather complex. Relatively expensive.
Blood biomarkers	Simple sampling procedure. Widespread use possible. Can test large numbers of biomarkers. Reproducible. Can serve as initial diagnostic examination in a more complex diagnostic procedure.	Relatively low concentrations of potential biomarkers due to the presence of the BBB. Unreliable findings because biomarkers may have other sources aside from the brain. Still low sensitivity and specificity of blood biomarkers.

Abbreviations: BBB—blood–brain barrier; CSF—cerebrospinal fluid; MRI—magnetic resonance imaging; PET—positron emission tomography.

**Table 5:** Studies that evaluated the use of plasma amyloid-β as biomarker for AD (adapted form Pais et al. [155]).

Population, Sample Size	Biomarker	Analytical Platform	Cut-Off/Threshold	Reference
397 participants (MissionAD1 and MissionAD2 trials.	Aβ_1-40_ Aβ_1-42_	ECL	Plasma Aβ_1-42_/Aβ_1-40_ ratio of 0.102 (AUC 0.94, sensitivity 96%, specificity 83.5%).	[159]
249 patients from the Plasma Test for Amyloidosis Risk Screening (PARIS) cohort + 437 patients form the MissionAD trial.	Plasma Aβ_1-40_ Plasma Aβ_1-42_	LC-MS	PARIS cohort: Plasma Aβ_1-42_/Aβ_1-40_ ratio ≥ 0.089 (AUC 0.79, sensitivity 85%, specificity 63%). Mission AD cohort: Plasma Aβ_1-42_/Aβ_1-40_ ratio ≥ 0.092 (AUC 0.86, sensitivity 90%, specificity 71%).	[160]
414 plasma samples from 6 independent US cohorts.	Plasma Aβ_1-40_ Plasma Aβ_1-42_	LC-MS	Plasma Aβ_1-42_/Aβ_1-40_ ratio ≥ 0.0975 (AUC 0.81).	[161]
317 participants from the Swedish BioFINDER-2 cohort.	Plasma Aβ_1-40_ Aβ_1-42_	ECL immunoassay	Plasma Aβ_1-42_/Aβ_1-40_ cut-off to differentiate between healthy and AD patients = 0.16. No data on accuracy, sensitivity, or specificity provided.	[162]

Abbreviations: AD—Alzheimer’s disease; ECL—electrochemiluminescence; LC-MS—liquid chromatography-tandem mass spectrometry.

**Table 6:** Studies that evaluated the use of plasma p-tau isoform levels as biomarker for AD (adapted form Pais et al. [155]).

Population, Sample Size	Biomarker	Analytical Platform	Cut-Off/Threshold	Reference
648 individuals (107 with MCI and 78 with AD) from the Stanford Aging and Memory study (SAMS) and the Stanford University Alzheimer’s Disease Research Center (ADRC) cohorts.	Plasma p-tau181.	Automated chemiluminescent enzyme immunoassay (Lumipulse).	p-tau181: 2.35 pg/mL (AUC 0.96, sensitivity 70.6%, specificity 93.3%).	[167]
234 individuals from the Translational Biomarkers in Aging and Dementia (TRIAD) cohort (60 MCI and 32 AD patients).	Plasma p-tau181 and p-tau231.	Simoa.	p-tau181: 15.085 pg/mL (AUC 0.96, sensitivity 87.5%, specificity 93.3%). p-tau231: 17.652 pg/mL (AUC 0.94, sensitivity 81.2%, specificity 93.3%).	[168]
A community-based cohort including 1329 participants (153 with MCI and 15 with dementia).	Plasma p-tau181 and p-tau217.	ECL-based assay.	p-tau181 ≥ 1.57 pg/mL (AUC 0.80). p-tau217 ≥ 0.25 pg/mL (AUC 0.85).	[63]
231 participants from the BioFINDER 2 cohort, of which 135 were cognitively unimpaired, and 96 were amyloid-PET positive, 18 being also tau-PET positive.	Plasma p-tau217.	Simoa and ECL.	Correlation with Aβ positivity: p-tau217 ≥ 0.07 pg/mL with Simoa (AUC 0.85, sensitivity 75%, specificity 85%). p-tau ≥ 0.26 pg/mL with ECL (AUC 0.88, sensitivity 84%, specificity 81%). Progression to AD dementia: p-tau ≥ 0.08 pg/mL with Simoa (AUC 0.88, sensitivity 81%). p-tau217 ≥ 0.31 pg/mL with ECL AUC 0.89, sensitivity 85%, specificity 84%).	[169]
397 participants, 22% with MCI and 21% with dementia.	Plasma p-tau217.	Simoa.	Correlation with positive Aβ-PET: p-tau217 ≥ 126.7 fg/mL (sensitivity 79%, specificity 89%). Identifying cognitively impaired participants: p-tau217 ≥ 126.7 fg/mL (sensitivity 82%, specificity 83%).	[170]
1189 participants from the Alzheimer’s Disease Neuroimaging Initiative (ADNI) cohort.	Plasma p-tau181.	Simoa.	p-tau181 ≥ 18.85 pg/mL (AUC 0.84, sensitivity 81.1%, specificity 81.6%).	[171]
144 patients recruited by the Centre for Memory Disorders at the University Hospital of Cologne, 54 with AD.	Plasma p-tau181.	Simoa.	p-tau181 ≥ 12.2 pg/mL (AUC 0.85, sensitivity 80%, specificity 79%).	[172]
317 participants from the Swedish BioFINDER-2 cohort.	Plasma p-tau181 and p-tau217.	ECL.	Thresholds: p-tau181: 7.48 pg/mL; p-tau217: 3.04 pg/mL.	[162]

Abbreviations: ECL—electrochemiluminescence; Simoa—digital ELISA with arrays of femtoliter-sized reaction chambers; AD—Alzheimer’s disease.

## Data Availability

No new data were created.

## References

[1] World Health Organization (2021). The Top 10 Causes of Death [Internet]. https://www.who.int/news-room/fact-sheets/detail/the-top-10-causes-of-death.

[2] Wimo A., Seeher K., Cataldi R., Cyhlarova E., Dielemann J.L., Frisell O., Guerchet M., Jönsson L., Malaha A.K., Nichols E. (2023). The worldwide costs of dementia in 2019. Alzheimers Dement..

[3] Scheltens P., De Strooper B., Kivipelto M., Holstege H., Chételat G., Teunissen C.E., Cummings J., van der Flier W.M. (2021). Alzheimer’s disease. Lancet.

[4] Hendriks S., Peetoom K., Bakker C., van der Flier W.M., Papma J.M., Koopmans R., Verhey F.R.J., de Vugt M., Köhler S., Young-Onset Dementia Epidemiology Study Group (2021). Global Prevalence of Young-Onset Dementia: A Systematic Review and Meta-analysis. JAMA Neurol..

[5] Rahimi J., Kovacs G.G. (2014). Prevalence of mixed pathologies in the aging brain. Alzheimers Res. Ther..

[6] Militaru M., Lighezan D.F., Tudoran C., Tudoran M., Militaru A.G. (2024). Factors Influencing the Development and Severity of Cognitive Decline in Patients with Chronic Heart Failure. Medicina.

[7] Militaru M., Lighezan D.F., Tudoran C., Zara F., Bucur A., Militaru A.G. (2025). Relationship Between Depression and Decreased Activity Level and Cognitive Impairment in Patients with Diabetes Mellitus Type 2 and/or Atrial Fibrillation. J. Clin. Med..

[8] Militaru M., Lighezan D.F., Tudoran C., Militaru A.G. (2024). Connections between Cognitive Impairment and Atrial Fibrillation in Patients with Diabetes Mellitus Type 2. Biomedicines.

[9] Livingston G., Huntley J., Liu K.Y., Costafreda S.G., Selbæk G., Alladi S., Ames D., Banerjee S., Burns A., Brayne C. (2024). Dementia prevention, intervention, and care: 2024 report of the Lancet standing Commission. Lancet.

[10] Nous A., Engelborghs S., Smolders I. (2021). Melatonin levels in the Alzheimer’s disease continuum: A systematic review. Alzheimers Res. Ther..

[11] Watroba M., Szukiewicz D. (2022). Sirtuins promote brain homeostasis, preventing Alzheimer’s disease through targeting neuroinflammation. Front. Physiol..

[12] Gottesman R.F., Albert M.S., Alonso A., Coker L.H., Coresh J., Davis S.M., Deal J.A., McKhann G.M., Mosley T.H., Sharrett A.R. (2017). Associations Between Midlife Vascular Risk Factors and 25-Year Incident Dementia in the Atherosclerosis Risk in Communities (ARIC) Cohort. JAMA Neurol..

[13] Pacholko A., Iadecola C. (2024). Hypertension, Neurodegeneration, and Cognitive Decline. Hypertension.

[14] Li Q.X., Gao H., Guo Y.X., Wang B.Y., Hua R.X., Gao L., Shang H.W., Lu X., Xu J.D. (2021). GLP-1 and Underlying Beneficial Actions in Alzheimer’s Disease, Hypertension, and NASH. Front. Endocrinol..

[15] Kim C.K., Lee Y.R., Ong L., Gold M., Kalali A., Sarkar J. (2022). Alzheimer’s Disease: Key Insights from Two Decades of Clinical Trial Failures. J. Alzheimers Dis..

[16] Bougea A., Gourzis P. (2024). Biomarker-Based Precision Therapy for Alzheimer’s Disease: Multidimensional Evidence Leading a New Breakthrough in Personalized Medicine. J. Clin. Med..

[17] Dubois B., Feldman H.H., Jacova C., Dekosky S.T., Barberger-Gateau P., Cummings J., Delacourte A., Galasko D., Gauthier S., Jicha G. (2007). Research criteria for the diagnosis of Alzheimer’s disease: Revising the NINCDS-ADRDA criteria. Lancet Neurol..

[18] Dubois B., Feldman H.H., Jacova C., Cummings J.L., Dekosky S.T., Barberger-Gateau P., Delacourte A., Frisoni G., Fox N.C., Galasko D. (2010). Revising the definition of Alzheimer’s disease: A new lexicon. Lancet Neurol..

[19] Dubois B., Villain N., Frisoni G.B., Rabinovici G.D., Sabbagh M., Cappa S., Bejanin A., Bombois S., Epelbaum S., Teichmann M. (2021). Clinical diagnosis of Alzheimer’s disease: Recommendations of the International Working Group. Lancet Neurol..

[20] Jack C.R., Andrews J.S., Beach T.G., Buracchio T., Dunn B., Graf A., Hansson O., Ho C., Jagust W., McDade E. (2024). Revised criteria for diagnosis and staging of Alzheimer’s disease: Alzheimer’s Association Workgroup. Alzheimers Dement..

[21] McKhann G., Drachman D., Folstein M., Katzman R., Price D., Stadlan E.M. (1984). Clinical diagnosis of Alzheimer’s disease: Report of the NINCDS-ADRDA Work Group under the auspices of Department of Health and Human Services Task Force on Alzheimer’s Disease. Neurology.

[22] McKhann G.M., Knopman D.S., Chertkow H., Hyman B.T., Jack C.R., Kawas C.H., Klunk W.E., Koroshetz W.J., Manly J.J., Mayeux R. (2011). The diagnosis of dementia due to Alzheimer’s disease: Recommendations from the National Institute on Aging-Alzheimer’s Association workgroups on diagnostic guidelines for Alzheimer’s disease. Alzheimers Dement..

[23] Dubois B., Feldman H.H., Jacova C., Hampel H., Molinuevo J.L., Blennow K., DeKosky S.T., Gauthier S., Selkoe D., Bateman R. (2014). Advancing research diagnostic criteria for Alzheimer’s disease: The IWG-2 criteria. Lancet Neurol..

[24] Dubois B., Hampel H., Feldman H.H., Scheltens P., Aisen P., Andrieu S., Bakardjian H., Benali H., Bertram L., Blennow K. (2016). Preclinical Alzheimer’s disease: Definition, natural history, and diagnostic criteria. Alzheimers Dement..

[25] Jack C.R., Bennett D.A., Blennow K., Carrillo M.C., Dunn B., Haeberlein S.B., Holtzman D.M., Jagust W., Jessen F., Karlawish J. (2018). Contributors. NIA-AA Research Framework: Toward a biological definition of Alzheimer’s disease. Alzheimers Dement..

[26] Young-Pearse T.L., Lee H., Hsieh Y.-C., Chou V., Selkoe D.J. (2023). Moving beyond amyloid and tau to capture the biological heterogeneity of Alzheimer’s disease. Trends Neurosci..

[27] Lista S., Mapstone M., Caraci F., Emanuele E., López-Ortiz S., Martín-Hernández J., Triaca V., Imbimbo C., Gabelle A., Mielke M.M. (2024). A critical appraisal of blood-based biomarkers for Alzheimer’s disease. Ageing Res. Rev..

[28] Hampel H., Cummings J., Blennow K., Gao P., Jack C.R., Vergallo A. (2021). Developing the ATX(N) classification for use across the Alzheimer’s disease continuum. Nat. Rev. Neurol..

[29] Molinuevo J.L., Ayton S., Batrla R., Bednar M.M., Bittner T., Cummings J., Fagan A.M., Hampel H., Mielke M.M., Mikulskis A. (2018). Current state of Alzheimer’s fluid biomarkers. Acta Neuropathol..

[30] Varesi A., Carrara A., Pires V.G., Floris V., Pierella E., Savioli G., Prasad S., Esposito C., Ricevuti G., Chirumbolo S. (2022). Blood-Based Biomarkers for Alzheimer’s Disease Diagnosis and Progression: An Overview. Cells.

[31] Jack C.R., Andrews S.J., Beach T.G., Buracchio T., Dunn B., Graf A., Hansson O., Ho C., Jagust W., McDade E. (2024). Revised criteria for the diagnosis and staging of Alzheimer’s disease. Nat. Med..

[32] Chong J.R., Ashton N.J., Karikari T.K., Tanaka T., Schöll M., Zetterberg H., Blennow K., Chen C.P., Lai M.K.P. (2021). Blood-based high sensitivity measurements of beta-amyloid and phosphorylated tau as biomarkers of Alzheimer’s disease: A focused review on recent advances. J. Neurol. Neurosurg. Psychiatry.

[33] Tikhonova M.A., Zhanaeva S.Y., Shvaikovskaya A.A., Olkov N.M., Aftanas L.I., Danilenko K.V. (2022). Neurospecific Molecules Measured in Periphery in Humans: How Do They Correlate with the Brain Levels? A Systematic Review. Int. J. Mol. Sci..

[34] Lam V., Takechi R., Hackett M.J., Francis R., Bynevelt M., Celliers L.M., Nesbit M., Mamsa S., Arfuso F., Das S. (2021). Synthesis of human amyloid restricted to liver results in an Alzheimer disease-like neurodegenerative phenotype. PLoS Biol..

[35] Liu Z.H., Wang Y.J., Bu X.L. (2023). Alzheimer’s disease: Targeting the peripheral circulation. Mol. Neurodegener..

[36] Zetterberg H. (2019). Blood-based biomarkers for Alzheimer’s disease—An update. J. Neurosci. Methods.

[37] Hampel H., O’Bryant S.E., Durrleman S., Younesi E., Rojkova K., Escott-Price V., Corvol J.-C., Broich K., Dubois B., Lista S. (2017). A Precision Medicine Initiative for Alzheimer’s Disease: The Road Ahead to Biomarker-Guided Integrative Disease Modeling. Climacteric.

[38] Jurcău M.C., Andronie-Cioara F.L., Jurcău A., Marcu F., Ţiț D.M., Pașcalău N., Nistor-Cseppentö D.C. (2022). The Link between Oxidative Stress, Mitochondrial Dysfunction and Neuroinflammation in the Pathophysiology of Alzheimer’s Disease: Therapeutic Implications and Future Perspectives. Antioxidants.

[39] Hansson O. (2021). Biomarkers for neurodegenerative diseases. Nat. Med..

[40] Constantinides V.C., Paraskevas G.P., Boufidou F., Bourbouli M., Pyrgelis E.-S., Stefanis L., Kapaki E. (2023). CSF Aβ42 and Aβ42/Aβ40 Ratio in Alzheimer’s Disease and Frontotemporal Dementias. Diagnostics.

[41] Amft M., Ortner M., Eichenlaub U., Goldhardt O., Diehl-Schmid J., Hedderich D.M., Yakushev I., Grimmer T. (2022). The cerebrospinal fluid biomarker ratio Aβ42/40 identifies amyloid positron emission tomography positivity better than Aβ42 alone in a heterogeneous memory clinic cohort. Alzheimers Res. Ther..

[42] Krawczuk D., Kulczyńska-Przybik A., Mroczko B. (2024). Clinical Application of Blood Biomarkers in Neurodegenerative Diseases—Present and Future Perspectives. Int. J. Mol. Sci..

[43] Watt A.D., Perez K.A., Rembach A.R., Masters C.L., Villemagne V.L., Barnham K.J. (2012). Variability in blood-based amyloid-beta assays: The need for consensus on pre-analytical processing. J. Alzheimers Dis..

[44] Leuzy A.S., Mattson-Carlgren N., Palmqvist S., Janelidze S., Gage J.L., Hansson O. (2022). Blood-based biomarkers for Alzheimer’s disease. EMBO Mol. Med..

[45] Olsson B., Lautner R., Andreasson U., Öhrfelt A., Portelius E., Bjerke M., Hölttä M., Rosén C., Olsson C., Strobel G. (2016). CSF and blood biomarkers for the diagnosis of Alzheimer’s disease: A systematic review and meta-analysis. Lancet Neurol..

[46] Schindler S.E., Bollinger J.G., Ovod V., Mawuenyega K.G., Li Y., Gordon B.A., Holtzman D.M., Morris J.C., Benzinger T.L.S., Xiong C. (2019). High-precision plasma β-amyloid 42/40 predicts current and future brain amyloidosis. Neurology.

[47] Teunissen C.E., Verberk I.M.W., Thijssen E.H., Vermunt L., Hansson O., Zetterberg H., van der Flier W.M., Mielke M.M., del Campo M. (2022). Blood-based biomarkers for Alzheimer’s disease: Towards clinical implementation. Lancet Neurol..

[48] Palmqvist S., Janelidze S., Stomrud E., Zetterberg H., Karl J., Zink K., Bittner T., Mattson N., Eichenlaub U., Blennow K. (2019). Performance of Fully Automated Plasma Assays as Screening Tests for Alzheimer’s Disease-Related-β-Amyloid Status. JAMA Neurol..

[49] Shea D., Colasurdo E., Smith A., Paschall C., Jayadev S., Keene C.D., Galasko D., Ko A., Li G., Peskind E. (2022). SOBA: Development and testing of a soluble oligomer binding assay for detection of amyloidogenic toxic oligomers. Proc. Natl. Acad. Sci. USA.

[50] Cullen N.C., Janelidze S., Mattsson-Carlgren N., Palmqvist S., Bittner T., Suridjan I., Jethwa A., Kollmorgen G., Brum W.S., Zetterberg H. (2023). Test-retest variability of plasma biomarkers in Alzheimer’s disease and its effects on clinical prediction models. Alzheimers Dement..

[51] Li Y., Schindler S.E., Bollinger J.G., Ovod V., Mawuenyega K.G., Weiner M.W., Shaw L.M., Masters C.L., Fowler C.J., Trojanowski J.Q. (2022). validation of plasma amyloid-β42/40 for detecting Alzheimer disease amyloid plaques. Neurology.

[52] Hu H., Bi Y.L., Shen X.N., Ma Y.H., Ou Y.N., Zhang W., Ma L.Z., Hu H.Y., Dong Q., Tan L. (2022). Application of the Amyloid/Tau/Neurodegeneration Framework in Cognitively Intact Adults: The CABLE Study. Ann. Neurol..

[53] Monane M., Johnson K.G., Snider B.J., Turner R.S., Drake J.D., Maraganore D.M., Bicksel J.L., Jacobs D.H., Ortega J.L., Henderson J. (2023). A blood biomarker test for brain amyloid impacts the clinical evaluation of cognitive impairment. Ann. Clin. Transl. Neurol..

[54] O’Connor A., Pannee J., Poole T., Arber C., Portelius E., Swift I.J., Heslegrave A.J., Abel E., Willumsen N., Rice H. (2021). Plasma amyloid-β ratios in autosomal dominant Alzheimer’s disease: The influence of genotype. Brain.

[55] Rawat P., Sehar U., Bisht J., Selman A., Culberson J., Reddy P.H. (2022). Phosphorylated Tau in Alzheimer’s Disease and Other Tauopathies. Int. J. Mol. Sci..

[56] Zhang H., Cao Y., Ma L., Wei Y., Li H. (2021). Possible Mechanisms of Tau Spread and Toxicity in Alzheimer’s Disease. Front. Cell Dev. Biol..

[57] Guo Y., Huang Y.-Y., Shen X.-N., Chen S.-D., Hu H., Wang Z.-T., Tan L., Yu J.-T. (2021). Characterization of Alzheimer’s Tau Biomarker Discordance using Plasma, CSF, and PET. Alzheimer’s Res. Ther..

[58] Toombs J., Zetterberg H. (2020). In the blood: Biomarkers for amyloid pathology and neurodegeneration in Alzheimer’s disease. Brain Commun..

[59] O’Connor A., Karikari T.K., Poole T., Ashton N.J., Lantero Rodriguez J., Khatun A., Swift I., Heslegrave A.J., Abel E., Chung E. (2021). Plasma phospho-tau181 in presymptomatic and symptomatic familial Alzheimer’s disease: A longitudinal cohort study. Mol. Psychiatry.

[60] Zetterberg H., Blennow K. (2020). Blood biomarkers: Democratizing Alzheimer’s diagnostics. Neuron.

[61] Bermudez C., Graff-Radford J., Syrjanen J.A., Stricker N.H., Algeciras-Schimnich A., Kouri N., Kremers W.K., Petersen R.C., Jack C.R., Knopman D.S. (2023). Plasma biomarkers for prediction of Alzheimer’s disease neuropathologic change. Acta Neuropathol..

[62] Janelidze S., Berron D., Smith R., Strandberg O., Proctor N.K., Dage J.L., Stomrud E., Palmqvist S., Mattsson-Carlgren N., Hansson O. (2021). Associations of Plasma Phospho-Tau217 Levels with Tau Positron Emission Tomography in Early Alzheimer Disease. JAMA Neurol..

[63] Mielke M.M., Dage J.L., Frank R.D., Algeciras-Schimnich A., Knopman D.S., Lowe V.J., Bu G., Vemuri P., Graff-Radford J., Jack C.R. (2022). Performance of plasma phosphorylated tau181 and 217 in the community. Nat. Med..

[64] Ashton N.J., Brum W.S., Di Molfetta G., Benedet A.L., Arsian B., Jonaitis E., Langhough R.E., Cody K., Wilson R., Carlsson C.M. (2024). Diagnostic Accuracy of a Plasma Phosphorylated Tau217 Immunoassay for Alzheimer Disase Pathology. JAMA Neurol..

[65] Mattson-Carlgren N., Salvadớ G., Ashton N.J., Tideman P., Stomrud E., Zetterberg H., Ossenkoppele R., Betthauser T., Cody K.A., Jonaitis E.M. (2023). Prediction of Longitudinal Cognitive Decline in Preclinical Alzheimer Disease Using Plasma Biomarkers. JAMA Neurol..

[66] Aguillon D., Langella S., Chen Y., Sanchez J.S., Su Y., Vila-Castelar C., Vasquez D., Zetterberg H., Hansson O., Dage J.L. (2023). Plasma p-tau217 predicts in vivo brain pathology and cognition in autosomal dominant Alzheimer’s disease. Alzheimers Dement..

[67] Saloner R., VandeVrede L., Asken B.M., Paolillo E.W., Gontrum E.Q., Wolf A., Lario-Lago A., Milà-Alomà M., Triana-Baltzer G., Kolb H.C. (2024). Plasma phosphorylated tau-217 exhibits sex-specific prognostication of cognitive decline and brain atrophy in cognitively unimpaired adults. Alzheimers Dement..

[68] Lehmann S., Schraen-Maschke S., Vidal J.S., Delaby C., Buee L., Blanc F., Paquet C., Allinquant B., Bombois S., Gabelle A. (2024). Clinical value of plasma ALZpath pTau217 immunoassay for assessing mild cognitive impairment. J. Neurol. Neurosurg. Psychiatry.

[69] Ashton N.J., Pascoal T.A., Karikari T.K., Benedet A.L., Lantero-Rodriguez J., Brinkmalm G., Snellman A., Schöll M., Troakes C., Hye A. (2021). Plasma p-tau231: A new biomarker for incipient Alzheimer’s disease pathology. Acta Neuropathol..

[70] Horie K., Salvadó G., Barthélemy N.R., Janelidze S., Li Y., He Y., Saef B., Chen C.D., Jiang H., Strandberg O. (2023). CSF MTBR-tau243 is a specific biomarker of tau tangle pathology in Alzheimer’s disease. Nat. Med..

[71] Horie K., Salvadó G., Koppisetti R.K., Janelidze S., Barthélemy N.R., He Y., Sato C., Gordon B.A., Jiang H., Benzinger T.L.S. (2025). Plasma MTBR-tau243 biomarker identifies tau tangle pathology in Alzheimer’s disease. Nat. Med..

[72] Chhatwal J.P., Schultz A.P., Dang Y., Ostaszewski B., Liu L., Yang H.S., Johnson K.A., Sperling R.A., Selkoe D.J. (2020). Plasma N-terminal tau fragment levels predict future cognitive decline and neurodegeneration in healthy elderly individuals. Nat. Commun..

[73] Khalil M., Teunissen C.E., Otto M., Piehl F., Sormani M.P., Gattringer T., Barro C., Kappos L., Comabella M., Fazekas F. (2018). Neurofilaments as biomarkers in neurological disorders. Nat. Rev. Neurol..

[74] Olsson B., Portelius E., Cullen N.C., Sandelius Å., Zetterberg H., Andreasson U., Höglund K., Irwin D.J., Grossman M., Weintraub D. (2019). Association of Cerebrospinal Fluid Neurofilament Light Protein Levels with Cognition in Patients with Dementia, Motor Neuron Disease, and Movement Disorders. JAMA Neurol..

[75] Jung Y., Damoiseaux J.S. (2024). The potential of blood neurofilament light as a marker of neurodegeneration for Alzheimer’s disease. Brain.

[76] Alcolea D., Beeri M.S., Rojas J.C., Gardner R.C., Lleó A. (2023). Blood Biomarkers in Neurodegenerative Diseases: Implications for the Clinical Neurologist. Neurology.

[77] Vrillon A., Ashton N.J., Karikari T.K., Götze K., Cognat E., Dumurgier J., Lilamand M., Zetterberg H., Blennow K., Paquet C. (2024). Comparison of CSF and plasma NfL and pNfH for Alzheimer’s disease diagnosis: A memory clinic study. J. Neurol..

[78] Gaetani L., Parnetti L., Calabresi P., Di Filippo M. (2021). Tracing Neurological Diseases in the Presymptomatic Phase: Insights from Neurofilament Light Chain. Front. Neurosci..

[79] De Meyer S., Blujdea E.R., Schaeverbeke J., Reinartz M., Luckett E.S., Adamczuk K., Van Laere K., Dupont P., Teunissen C.E., Vandenberghe R. (2024). Longitudinal associations of serum biomarkers with early cognitive, amyloid and grey matter changes. Brain.

[80] Chatterjee P., Pedrini S., Doecke J.D., Thota R., Villemagne V.L., Doré V., Singh A.K., Wang P., Rainey-Smith S., Fowler C. (2023). Plasma Aβ42/40 ratio, p-tau181, GFAP, and NfL across the Alzheimer’s disease continuum: A cross-sectional and longitudinal study in the AIBL cohort. Alzheimers Dement..

[81] Nabizadeh F., Balabandian M., Rostami M.R., Kankam S.B., Ranjbaran F., Pourhamzeh M., Alzheimer’s Disease Neuroimaging Initiative (ADNI) (2022). Plasma neurofilament light levels correlate with white matter damage prior to Alzheimer’s disease: Results from ADNI. Aging Clin. Exp. Res..

[82] Hofmann A., Haesler L.M., Preische O., Gräber-Sultan S., Obermüller U., Vöglein J., Levin J., Laske C., Fitzpatrick C.D., Levin R. (2023). Refinement of Neurofilament light Dynamics in CSF and Blood for familial Alzheimer’s Disease. Alzheimer’s Dement..

[83] Jurcau M.C., Jurcau A., Diaconu R.G., Hogea V.O., Nunkoo V.S. (2024). A Systematic Review of Sporadic Creutzfeldt-Jakob Disease: Pathogenesis, Diagnosis, and Therapeutic Attempts. Neurol. Int..

[84] Kang Y., Feng Z., Zhang Q., Liu M., Li Y., Yang H., Zheng L., Cheng C., Zhou W., Lou D. (2025). Identification of circulating biomarkers for cognitive decline in a large community-based population in Chongqing China. Alzheimers Dement..

[85] Vila-Castelar C., Chen Y., Langella S., Lopera F., Zetterberg H., Hansson O., Dage J.L., Janelidzde S., Su Y., Chen K. (2023). Sex differences in blood biomarkers and cognitive performance in individuals with autosomal dominant Alzheimer’s disease. Alzheimers Dement..

[86] Fitzgerald K.C., Sotirchos E.S., Smith M.D., Lord H.N., DuVal A., Mowry E.M., Calabresi P.A. (2022). Contributors to Serum NfL Levels in People without Neurologic Disease. Ann. Neurol..

[87] Jurcau M.C., Jurcau A., Cristian A., Hogea V.O., Diaconu R.G., Nunkoo V.S. (2024). Inflammaging and Brain Aging. Int. J. Mol. Sci..

[88] Andronie-Cioara F.L., Ardelean A.I., Nistor-Cseppento C.D., Jurcau A., Jurcau M.C., Pascalau N., Marcu F. (2023). Molecular Mechanisms of Neuroinflammation in Aging and Alzheimer’s Disease Progression. Int. J. Mol. Sci..

[89] Bellaver B., Ferrari-Souza J.P., Uglione da Ros L., Carter S.F., Rodriguez-Vieitez E., Nordberg A., Pellerin L., Rosa-Neto P., Leffa D.T., Zimmer E.R. (2021). Astrocyte Biomarkers in Alzheimer Disease: A Systematic Review and Meta-analysis. Neurology.

[90] Benedet A.L., Milà-Alomà M., Vrillon A., Ashton N.J., Pascoal T.A., Lussier F., Karikari T.K., Hourregue C., Cognat E., Dumurgier J. (2021). Differences Between Plasma and Cerebrospinal Fluid Glial Fibrillary Acidic Protein Levels Across the Alzheimer Disease Continuum. JAMA Neurol..

[91] Stocker H., Beyer L., Perna L., Rujescu D., Holleczek B., Beyreuther K., Stockmann J., Schöttker B., Gerwert K., Brenner H. (2023). Association of plasma biomarkers, p-tau181, glial fibrillary acidic protein, and neurofilament light, with intermediate and long-term clinical Alzheimer’s disease risk: Results from a prospective cohort followed over 17 years. Alzheimers Dement..

[92] Snellman A., Ekblad L.L., Ashton N.J., Karikari T.K., Lantero-Rodriguez J., Pietilä E., Koivumäki M., Helin S., Karrasch M., Zetterberg H. (2023). Head-to-head comparison of plasma p-tau181, p-tau231 and glial fibrillary acidic protein in clinically unimpaired elderly with three levels of APOE4-related risk for Alzheimer’s disease. Neurobiol. Dis..

[93] Park M.-K., Ahn J., Kim Y.-J., Lee J.-W., Lee J.-C., Hwang S.-J., Kim K.-C. (2024). Predicting Longitudinal Cognitive Decline and Alzheimer’s Conversion in Mild Cognitive Impairment Patients Based on Plasma Biomarkers. Cells.

[94] Bellaver B., Povala G., Ferreira P.C.L., Ferrari-Souza J.P., Leffa D.T., Lussier F.Z., Benedet A.L., Ashton N.J., Triana-Baltzer G., Kolb H.C. (2023). Astrocyte reactivity influences amyloid-β effects on tau pathology in preclinical Alzheimer’s disease. Nat. Med..

[95] Asken B.M., Elahi F.M., La Joie R., Strom A., Staffaroni A.M., Lindbergh C.A., Apple A.C., You M., Weiner-Light S., Brathaban N. (2020). Plasma Glial Fibrillary Acidic Protein Levels Differ Along the Spectra of Amyloid Burden and Clinical Disease Stage. J. Alzheimers Dis..

[96] Oeckl P., Halbgebauer S., Anderl-Straub S., Steinacker P., Huss A.M., Neugebauer H., von Arnim C.A.F., Diehl-Schmid J., Grimmer T., Kornhuber J. (2019). Glial Fibrillary Acidic Protein in Serum is Increased in Alzheimer’s Disease and Correlates with Cognitive Impairment. J. Alzheimers Dis..

[97] Chouliaras L., Thomas A., Malpetti M., Donaghy P., Kane J., Mak E., Savulich G., Prats-Sedano M.A., Heslegrave A.J., Zetterberg H. (2022). Differential levels of plasma biomarkers of neurodegeneration in Lewy body dementia, Alzheimer’s disease, frontotemporal dementia and progressive supranuclear palsy. J. Neurol. Neurosurg. Psychiatry.

[98] Morenas-Rodríguez E., Li Y., Nuscher B., Franzmeier N., Xiong C., Suárez-Calvet M., Fagan A.M., Schultz S., Gordon B.A., Benzinger T.L.S. (2022). Soluble TREM2 in CSF and its association with other biomarkers and cognition in autosomal-dominant Alzheimer’s disease: A longitudinal observational study. Lancet Neurol..

[99] La Rosa F., Agostini S., Piancone F., Marventano I., Hernis A., Fenoglio C., Galimberti D., Scarpini E., Saresella M., Clerici M. (2023). TREM2 Expression and Amyloid-Beta Phagocytosis in Alzheimer’s Disease. Int. J. Mol. Sci..

[100] Hu N., Tan M.-S., Yu J.-T., Sun L., Tan L., Wang Y.-L., Jiang T., Tan L. (2013). Increased Expression of TREM2 in Peripheral Blood of Alzheimer’s Disease Patients. J. Alzheimer’s Dis..

[101] Wang Q., Xu Y., Qi C., Liu A., Zhao Y. (2020). Association Study of Serum Soluble TREM2 with Vascular Dementia in Chinese Han Population. Int. J. Neurosci..

[102] Hok-A-Hin Y.S., Del Campo M., Boiten W.A., Stoops E., Vanhooren M., Lemstra A.W., van der Flier W.M., Teunissen C.E. (2023). Neuroinflammatory CSF biomarkers MIF, sTREM1, and sTREM2 show dynamic expression profiles in Alzheimer’s disease. J. Neuroinflammation.

[103] Vergallo A., Lista S., Lemercier P., Chiesa P.A., Zetterberg H., Blennow K., Potier M.C., Habert M.O., Baldacci F., Cavedo E. (2020). Association of plasma YKL-40 with brain amyloid-β levels, memory performance, and sex in subjective memory complainers. Neurobiol. Aging.

[104] Ferrari-Souza J.P., Ferreira P.C.L., Bellaver B., Tissot C., Wang Y.T., Leffa D.T., Brum W.S., Benedet A.L., Ashton N.J., De Bastiani M.A. (2022). Astrocyte biomarker signatures of amyloid-β and tau pathologies in Alzheimer’s disease. Mol. Psychiatry.

[105] Wilczyńska K., Maciejczyk M., Zalewska A., Waszkiewicz N. (2021). Serum Amyloid Biomarkers, Tau Protein and YKL-40 Utility in Detection, Differential Diagnosing, and Monitoring of Dementia. Front. Psychiatry.

[106] Thomas A., Guo J., Reyes-Dumeyer D., Sanchez D., Scarmeas N., Manly J.J., Brickman A.M., Lantigua R.A., Mayeux R., Gu Y. (2025). Inflammatory biomarkers profiles and cognition among older adults. Sci. Rep..

[107] Swardfager W., Lanctôt K., Rothenburg L., Wong A., Cappell J., Herrmann N. (2010). A Meta-Analysis of Cytokines in Alzheimer’s Disease. Biol. Psychiatry.

[108] Lai K.S.P., Liu C.S., Rau A., Lanctôt K.L., Köhler C.A., Pakosh M., Carvalho A.F., Herrmann N. (2017). Peripheral Inflammatory Markers in Alzheimer’s Disease: A Systematic Review and Meta-Analysis of 175 Studies. J. Neurol. Neurosurg. Psychiatry.

[109] Ng A., Tam W.W., Zhang M.W., Ho C.S., Husain S.F., McIntyre R.S., Ho R.C. (2018). IL-1β, IL-6, TNF- α and CRP in Elderly Patients with Depression or Alzheimer’s Disease: Systematic Review and Meta-Analysis. Sci. Rep..

[110] Foley K.E., Winder Z., Sudduth T.L., Martin B.J., Nelson P.T., Jicha G.A., Harp J.P., Weekman E.M., Wilcock D.M. (2024). Alzheimer’s disease and inflammatory biomarkers positively correlate in plasma in the UK-ADRC cohort. Alzheimers Dement..

[111] Liang C.-S., Tsai C.-L., Lin G.-Y., Lee J.-T., Lin Y.-K., Chu C.-S., Sung Y.-F., Tsai C.-K., Yeh T.-C., Chu H.-T. (2021). Better Identification of Cognitive Decline with Interleukin-2 than with Amyloid and Tau Protein Biomarkers in Amnestic Mild Cognitive Impairment. Front. Aging Neurosci..

[112] Chakrabarty P., Li A., Ceballos-Diaz C., Eddy J.A., Funk C.C., Moore B., DiNunno N., Rosario A.M., Cruz P.E., Verbeeck C. (2015). IL-10 alters immunoproteostasis in APP mice, increasing plaque burden and worsening cognitive behavior. Neuron.

[113] Ji D., Chen W.Z., Zhang L., Zhang Z.H., Chen L.J. (2024). Gut microbiota, circulating cytokines and dementia: A Mendelian randomization study. J. Neuroinflammation.

[114] Hampel H., Nisticò R., Seyfried N.T., Levey A.I., Modeste E., Lemercier P., Baldacci F., Toschi N., Garaci F., Perry G. (2021). Omics sciences for systems biology in Alzheimer’s disease: State-of-the-art of the evidence. Ageing Res. Rev..

[115] Jiang Y., Zhou X., Ip F.C., Chan P., Chen Y., Lai N.C.H., Cheung K., Lo R.M.N., Tong E.P.S., Wong B.W.Y. (2022). Large-scale plasma proteomic profiling identifies a high-performance biomarker panel for Alzheimer’s disease screening and staging. Alzheimers Dement..

[116] Lacar B., Ferdosi S., Alavi A., Stukalov A., Venkataraman G.R., de Geus M., Dodge H., Wu C.Y., Kivisakk P., Das S. (2024). Identification of Novel Biomarkers for Alzheimer’s Disease and Related Dementias Using Unbiased Plasma Proteomics. bioRxiv.

[117] Huang Y.-L., Chang W.-J., Huang C.-H., Lin C.-H., Peng L.-N., Chung C.-P., Chen L.-K., Lee W.-J. (2025). Proteo-metabolomic insights for early dual physical and cognitive impairments: A search for biomarkers of healthy aging based on muscle-brain crosstalk. Aging Cell.

[118] Jurcau A., Jurcau M.C. (2022). Therapeutic Strategies in Huntington’s Disease: From Genetic Defect to Gene Therapy. Biomedicines.

[119] Yao Q., Chen Y., Zhou X. (2019). The roles of microRNAs in epigenetic regulation. Curr. Opin. Chem. Biol..

[120] Wang L., Shui X., Diao Y., Chen D., Zhou Y., Lee T.H. (2023). Potential Implications of miRNAs in the Pathogenesis, Diagnosis, and therapeutics of Alzheimer’s Disease. Int. J. Mol. Sci..

[121] Denk J., Oberhauser F., Kornhuber J., Wiltfang J., Fassbender K., Schroeter M.L., Volk A.E., Diehl-Schmid J., Prudlo J., Danek A. (2018). Specific serum and CSF microRNA profiles distinguish sporadic behavioural variant of frontotemporal dementia compared with Alzheimer patients and cognitively healthy controls. PLoS ONE.

[122] Siedlecki-Wullich D., Miñano-Molina A.J., Rodríguez-Álvarez J. (2021). microRNAs as Early Biomarkers of Alzheimer’s Disease: A Synaptic Perspective. Cells.

[123] Kenny A., McArdle H., Calero M., Rabano A., Madden S.F., Adamson K., Forster R., Spain E., Prehn J.H.M., Henshall D.C. (2019). Elevated Plasma microRNA-206 Levels Predict Cognitive Decline and Progression to Dementia from Mild Cognitive Impairment. Biomolecules.

[124] Ansari A., Maffioletti E., Milanesi E., Marizzoni M., Frisoni G.B., Blin O., Richardson J.C., Bordet R., Forloni G., Gennarelli M. (2019). miR-146a and miR-181a are involved in the progression of mild cognitive impairment to Alzheimer’s disease. Neurobiol. Aging.

[125] Liu S., Park T., Krüger D.M., Pena-Centeno T., Burkhardt S., Schutz A.-L., Huang Y.-N., Rosewood T., Chaudhuri S., Cho M.Y. (2024). Plasma miRNAs across the Alzheimer’s disease continuum: Relationship to central biomarkers. Alzheimer’s Dement..

[126] Krüger D.M., Pena-Centeno T., Liu S., Park T., Kaurani L., Pradhan R., Huang Y.-N., Risacher S.L., Burkhardt S., Schütz A.-L. (2024). The plasma miRNAome in ADNI: Signatures to aid the detection of at-risk individuals. Alzheimer’s Dement..

[127] Gupta N., Jadhav S., Tan K.L., Saw G., Mallilankaraman K.B., Dheen S.T. (2020). miR-142-3p regulates BDNF expression in activated rodent microglia through its target CMAK2A. Front. Cell. Neurosci..

[128] Zhang L., Dong H., Si Y., Wu N., Cao H., Mei B., Meng B. (2019). miR-125b promotes tau phosphorylation by targeting the neural cell adhesion molecule in neuropathological progression. Neurobiol. Aging.

[129] Absalon S., Kochanek D.M., Raghavan V., Krichevsky A.M. (2013). MiR-26b, upregulated in Alzheimer’s disease, activates cell cycle entry, tau phosphorylation, and apoptosis in postmitotic neurons. J. Neurosci..

[130] Yin C., Liufu C., Ye S., Zhu T., Jiang J., Wang M., Zhou L., Yao L., Wang Y., Shi B. (2025). Tumor-derived exosomal *KPNA2* activates fibroblasts and interacts with KIFC1 to promote bladder cancer progression, a process inhibited by miR-26b-5p. Cell Mol. Biol. Lett..

[131] Xiao Y., Zheng S., Duan N., Li X., Wen J. (2021). MicroRNA-26b-5p alleviates cerebral ischemia-reperfusion injury in rats via inhibiting the N-myc/PTEN axis by downregulating KLF10 expression. Hum. Exp. Toxicol..

[132] Kalluri R., LeBleu V.S. (2020). The biology, function, and biomedical applications of exosomes. Science.

[133] Eren E., Hunt J.F.V., Shardell M., Chawla S., Tran J., Gu J., Vogt N.M., Johnson S.C., Bendlin B.B., Kapogiannis D. (2020). Extracellular vesicle biomarkers of Alzheimer’s disease associated with sub-clinical cognitive decline in late middle age. Alzheimers Dement..

[134] Nistor-Cseppentö D.C., Jurcău M.C., Jurcău A., Andronie-Cioară F.L., Marcu F. (2022). Stem Cell- and Cell-Based Therapies for Ischemic Stroke. Bioengineering.

[135] Banks W.A., Sharma P., Bullock K.M., Hansen K.M., Ludwig N., Whiteside T.L. (2020). Transport of Extracellular Vesicles across the Blood-Brain Barrier: Brain Pharmacokinetics and Effects of Inflammation. Int. J. Mol. Sci..

[136] Delgado-Peraza F., Nogueras-Ortiz C.J., Volpert O., Liu D., Goetzl E.J., Mattson M.P., Greig N.H., Eitan E., Kapogiannis D. (2021). Neuronal and Astrocytic Extracellular Vesicle Biomarkers in Blood Reflect Brain Pathology in Mouse Models of Alzheimer’s Disease. Cells.

[137] Cai H., Pang Y., Wang Q., Qin W., Wei C., Li Y., Li T., Li F., Wang Q., Li Y. (2022). Proteomic profiling of circulating plasma exosomes reveals novel biomarkers of Alzheimer’s disease. Alzheimer’s Res. Ther..

[138] Kalra H., Adda C.G., Liem M., Ang C.S., Mechler A., Simpson R.J., Hulett M.D., Mathivanan S. (2013). Comparative proteomics evaluation of plasma exosome isolation techniques and assessment of the stability of exosomes in normal human blood plasma. Proteomics.

[139] Eitan E., Thornton-Wells T., Elgart K., Erden E., Gershun E., Levine A., Volpert O., Azadeh M., Smith D.G., Kapogiannis D. (2023). Synaptic proteins in neuron-derived extracellular vesicles as biomarkers for Alzheimer’s disease: Novel methodology and clinical proof of concept. Extracell. Vesicles Circ. Nucleic Acids..

[140] Fiandaca M.S., Kapogiannis D., Mapstone M., Boxer A., Eitan E., Schwartz J.B., Abner E.L., Petersen R.C., Federoff H.J., Miller B.L. (2015). Identification of preclinical Alzheimer’s disease by a profile of pathogenic proteins in neurally derived blood exosomes: A case-control study. Alzheimers Dement..

[141] Kumari S., Dhapola R., Reddy D.H. (2023). Apoptosis in Alzheimer’s disease: Insight into the signaling pathways and therapeutic avenues. Apoptosis.

[142] Nakamura M., Li Y., Choi B.R., Matas-Rico E., Troncoso J., Takahashi C., Sockanathan S. (2021). GDE2-RECK controls ADAM10 α-secretase-mediated cleavage of amyloid precursor protein. Sci. Transl. Med..

[143] McMillan N., Kirschen G.W., Desai S., Xia E., Tsirka S.E., Aguirre A. (2022). ADAM10 facilitates rapid neural stem cell cycling and proper positioning within the subventricular zone niche via JAMC/RAP1Gap signaling. Neural Regen. Res..

[144] Liu W.-L., Lin H.-W., Lin M.-R., Yu Y., Liu H.-H., Dai Y.-L., Chen L.-W., Jia W.-W., He X.-J., Li X.-L. (2022). Emerging blood exosome-based biomarkers for preclinical and clinical Alzheimer’s disease: A meta-analysis and systematic review. Neural Regen. Res..

[145] Jia L., Zhu M., Kong C., Pang Y., Zhang H., Qiu Q., Wei C., Tang Y., Wang Q., Li Y. (2021). Blood neuro-exosomal synaptic proteins predict Alzheimer’s disease at the asymptomatic stage. Alzheimers Dement..

[146] Visconte C., Fenoglio C., Serpente M., Muti P., Sacconi A., Rigoni M., Arighi A., Borracci V., Arcaro M., Arosio B. (2023). Altered Extracellular Vesicle miRNA Profile in Prodromal Alzheimer’s Disease. Int. J. Mol. Sci..

[147] Guimarães T.R., Swanson E., Kofler J., Thathiah A. (2021). G protein-coupled receptor kinases are associated with Alzheimer’s disease pathology. Neuropathol. Appl. Neurobiol..

[148] Wegmann S., Biernat J., Mandelkow E. (2021). A current view on Tau protein phosphorylation in Alzheimer’s disease. Curr. Opin. Neurobiol..

[149] Wang Y., Yuan P., Ding L., Zhu J., Qi X., Zhang Y., Li Y., Xia X., Zheng J.C. (2022). Circulating extracellular vesicle-containing microRNAs reveal potential pathogenesis of Alzheimer’s disease. Front. Cell. Neurosci..

[150] Kaštelan S., Braš M., Pjevač N., Bakija I., Tomić Z., Pjevač Keleminić N., Gverović Antunica A. (2023). Tear Biomarkers and Alzheimer’s Disease. Int. J. Mol. Sci..

[151] Freeman S.H., Raju S., Hyman B.T., Frosch M.P., Irizarry M.C. (2007). Plasma Abeta levels do not reflect brain Abeta levels. J. Neuropathol. Exp. Neurol..

[152] Rissin D.M., Kan C.W., Campbell T.G., Howes S.C., Fournier D.R., Song L., Piech T., Patel P.P., Chang L., Rivnak A.J. (2010). Single-molecule enzyme-linked immunosorbent assay detects serum proteins at subfemtomolar concentrations. Nat. Biotechnol..

[153] Herskovits A.Z., Locascio J.J., Peskind E.R., Li G., Hyman B.T. (2013). A Luminex assay detects amyloid β oligomers in Alzheimer’s disease cerebrospinal fluid. PLoS ONE.

[154] Song F., Poljak A., Valenzuela M., Mayeux R., Smythe G.A., Sachdev P.S. (2011). Meta-analysis of plasma amyloid-beta levels in Alzheimer’s disease. J. Alzheimers Dis..

[155] Pais M.V., Forlenza O.V., Diniz B.S. (2023). Plasma Biomarkers of Alzheimer’s Disease: A Review of Available Assays, Recent Developments, and Implications for Clinical Practice. J. Alzheimers Dis. Rep..

[156] Janelidze S., Bali D., Ashton N.J., Barthélemy N.R., Vanbrabant J., Stoops E., Vanmechelen E., He Y., Dolado A.O., Triana-Baltzer G. (2023). Head-to-head comparison of 10 plasma phospho-tau assays in prodromal Alzheimer’s disease. Brain.

[157] Verberk I.M.W., Hendriksen H.M.A., van Harten A.C., Wesselman L.M.P., Verfaillie S.C.J., van den Bosch K.A., Slot R.E.R., Prins N.D., Scheltens P., Teunissen C.E. (2020). Plasma amyloid is associated with the rate of cognitive decline in cognitively normal elderly: The SCIENCe project. Neurobiol. Aging.

[158] Janelidze S., Teunissen C.E., Zetterberg H., Allué J.A., Sarasa L., Eichenlaub U., Bittner T., Ovod V., Verberk I.M.W., Toba K. (2021). Head-to-Head Comparison of 8 Plasma Amyloid-β 42/40 Assays in Alzheimer Disease. JAMA Neurol..

[159] Yamashita K., Miura M., Watanabe S., Ishiki K., Arimatsu Y., Kawahira J., Kubo T., Sasaki K., Arai T., Hagino K. (2022). Fully automated and highly specific plasma β-amyloid immunoassays predict β-amyloid status defined by amyloid positron emission tomography with high accuracy. Alzheimers Res. Ther..

[160] Hu Y., Kirmess K.M., Meyer M.R., Rabinovici G.D., Gatsonis C., Siegel B.A., Whitmer R.A., Apgar C., Hanna L., Kanekiyo M. (2022). Assessment of a Plasma Amyloid Probability Score to Estimate Amyloid Positron Emission Tomography Findings Among Adults with Cognitive Impairment. JAMA Netw. Open..

[161] West T., Kirmess K.M., Meyer M.R., Holubasch M.S., Knapik S.S., Hu Y., Contois J.H., Jackson E.N., Harpstrite S.E., Bateman R.J. (2021). A blood-based diagnostic test incorporating plasma Aβ42/40 ratio, ApoE proteotype, and age accurately identifies brain amyloid status: Findings from a multi cohort validity analysis. Mol. Neurodegener..

[162] Pereira J.B., Janelidze S., Stomrud E., Palmqvist S., van Westen D., Dage J.L., Mattsson-Carlgren N., Hansson O. (2021). Plasma markers predict changes in amyloid, tau, atrophy and cognition in non-demented subjects. Brain.

[163] Tanner J.A., Rabinovici G.D. (2021). Relationship Between Tau and Cognition in the Evolution of Alzheimer’s Disease: New Insights from Tau PET. J. Nucl. Med..

[164] Hardy J., Selkoe D.J. (2002). The amyloid hypothesis of Alzheimer’s disease: Progress and problems on the road to therapeutics. Science.

[165] Chen Y.R., Liang C.S., Chu H., Voss J., Kang X.L., O’Connell G., Jen H.J., Liu D., Shen Hsiao S.T., Chou K.R. (2021). Diagnostic accuracy of blood biomarkers for Alzheimer’s disease and amnestic mild cognitive impairment: A meta-analysis. Ageing Res. Rev..

[166] Palmqvist S., Tideman P., Cullen N., Zetterberg H., Blennow K., Dage J.L., Stomrud E., Janelidze S., Mattsson-Carlgren N., Alzheimer’s Disease Neuroimaging Initiative (2021). Prediction of future Alzheimer’s disease dementia using plasma phospho-tau combined with other accessible measures. Nat. Med..

[167] Wilson E.N., Young C.B., Ramos Benitez J., Swarovski M.S., Feinstein I., Vandijck M., Le Guen Y., Kasireddy N.M., Shahid M., Corso N.K. (2022). Performance of a fully-automated Lumipulse plasma phospho-tau181 assay for Alzheimer’s disease. Alzheimers Res. Ther..

[168] Tissot C., Therriault J., Kunach P., Benedet A.L., Pascoal T.A., Ashton N.J., Karikari T.K., Servaes S., Lussier F.Z., Chamoun M. (2022). Comparing tau status determined via plasma pTau181, pTau231 and [^18^F]MK6240 tau-PET. EBioMedicine.

[169] Groot C., Smith R., Stomrud E., Binette A.P., Leuzy A., Wuestefeld A., Wisse L.E.M., Palmqvist S., Mattsson-Carlgren N., Janelidze S. (2023). Phospho-tau with subthreshold tau-PET predicts increased tau accumulation rates in amyloid-positive individuals. Brain.

[170] Doré V., Doecke J.D., Saad Z.S., Triana-Baltzer G., Slemmon R., Krishnadas N., Bourgeat P., Huang K., Burnham S., Fowler C. (2022). Plasma p217+tau versus NAV4694 amyloid and MK6240 tau PET across the Alzheimer’s continuum. Alzheimers Dement..

[171] Shen X.N., Huang Y.Y., Chen S.D., Guo Y., Tan L., Dong Q., Yu J.T., Alzheimer’s Disease Neuroimaging Initiative (2021). Plasma phosphorylated-tau181 as a predictive biomarker for Alzheimer’s amyloid, tau and FDG PET status. Transl. Psychiatry.

[172] Gerards M., Schild A.K., Meiberth D., Rostamzadeh A., Vehreschild J.J., Wingen-Heimann S., Johannis W., Martino Adami P., Onur O.A., Ramirez A. (2022). Alzheimer’s Disease Plasma Biomarkers Distinguish Clinical Diagnostic Groups in Memory Clinic Patients. Dement. Geriatr. Cogn. Disord..

[173] Roses A., Saunders A., Corder E.H., Risch N., Haines J., Pericak-Vance M., Han S.H., Einstein G., Hulette C., Schmechel D. (1995). Apolipoprotein E4 and E2 are susceptibility genes that affect the rate of Alzheimer disease expressivity. J. Cell. Biochem..

[174] Palmqvist S., Janelidze S., Quiroz Y.T., Zetterberg H., Lopera F., Stomrud E., Su Y., Chen Y., Serrano G.E., Leuzy A. (2020). Discriminative Accuracy of Plasma Phospho-tau217 for Alzheimer Disease vs Other Neurodegenerative Disorders. JAMA.

[175] Chatterjee P., Pedrini S., Ashton N.J., Tegg M., Goozee K., Singh A.K., Karikari T.K., Simrén J., Vanmechelen E., Armstrong N.J. (2022). Diagnostic and prognostic plasma biomarkers for preclinical Alzheimer’s disease. Alzheimers Dement..

[176] Asken B.M., VandeVrede L., Rojas J.C., Fonseca C., Staffaroni A.M., Elahi F.M., Lindbergh C.A., Apple A.C., You M., Weiner-Light S. (2022). Lower White Matter Volume and Worse Executive Functioning Reflected in Higher Levels of Plasma GFAP among Older Adults with and Without Cognitive Impairment. J. Int. Neuropsychol. Soc..

[177] Oeckl P., Anderl-Straub S., Von Armin C.A.F., Baldeiras I., Diehl-Schmid J., Grimmer T., Halbgebauer S., Kort A.M., Lima M., Marques T.M. (2022). Serum GFAP differentiates Alzheimer’s disease from frontotemporal dementia and predicts MCI-to-dementia conversion. J. Neurol. Neurosurg. Psychiatry.

[178] Hansson O., Edelmayer R.M., Boxer A.L., Carillo M.C., Mielke M.M., Rabinovici G.D., Salloway S., Sperling R., Zetterberg K., Teunissen C.E. (2022). The Alzheimer’s Association appropriate use recommendations for blood biomarkers in Alzheimer’s disease. Alzheimers Dement..

[179] Wu J., Xiao Z., Wang M., Wu W., Ma X., Liang X., Zheng L., Ding S., Luo J., Cao Y. (2024). The impact of kidney function on plasma neurofilament light and phospho-tau181 in a community-based cohort: The Shanghai Aging Study. Alzheimers Res. Ther..

[180] Ashton N.J., Keshavan A., Brum W.S., Andreasson U., Arslan B., Droescher M., Barghorn S., Vanbrabant J., Lambrechts C., Van Loo M. (2025). The Alzheimer’s Association Global Biomarker Standardization Consortium (GBSC) plasma phospho-tau Round Robin study. Alzheimers Dement..

[181] Ibanez L., Liu M., Beric A., Timsina J., Kohlfeld P., Bergmann K., Lowery J., Sykora N., Sanchez-Montejo B., Brock W. (2025). Benchmarking of a multi-biomarker low-volume panel for Alzheimer’s disease and related dementia research. Alzheimers Dement..

[182] Mielke M.M., Fowler N.R. (2024). Alzheimer disease blood biomarkers: Considerations for population-level use. Nat. Rev. Neurol..

[183] Nunkoo V.S., Cristian A., Jurcau A., Diaconu R.G., Jurcau M.C. (2024). The Quest for Eternal Youth: Hallmarks of Aging and Rejuvenating Therapeutic Strategies. Biomedicines.

[184] Justice J.N., Leng X.I., LeBrasseur N.K., Tchkonia T., Kirkland J.L., Mitin N., Liu Y., Kritchevsky S.B., Nicklas B.J., Ding J. (2024). Caloric Restriction Intervention Alters Specific Circulating Biomarkers of the Senescence-Associated Secretome in Middle-Aged and Older Adults with Obesity and Prediabetes in an 18-Week Randomized Controlled Trial. J. Gerontol. A Biol. Sci. Med. Sci..

[185] Rabinovici G.D., Gatsonis C., Apgar C., Chaudhary K., Gareen I., Hanna L., Hendrix J., Hillner B.E., Olson C., Lesman-Segev O.H. (2019). Association of Amyloid Positron Emission Tomography with Subsequent Change in Clinical Management Among Medicare Beneficiaries with Mild Cognitive Impairment or Dementia. JAMA.

[186] Suemoto C.K., Ferretti-Rebustini R.E., Rodriguez R.D., Leite R.E., Soterio L., Brucki S.M., Spera R.R., Cippiciani T.M., Farfel J.M., Chiavegatto Filho A. (2017). Neuropathological diagnoses and clinical correlates in older adults in Brazil: A cross-sectional study. PLoS Med..

[187] Milà-Alomà M., Ashton N.J., Shekari M., Salvadó G., Ortiz-Romero P., Montoliu-Gaya L., Benedet A.L., Karikari T.K., Lantero-Rodriguez J., Vanmechelen E. (2022). Plasma p-tau231 and p-tau217 as state markers of amyloid-β pathology in preclinical Alzheimer’s disease. Nat. Med..

[188] Palmqvist S., Stomrud E., Cullen N., Janelidze S., Manuilova E., Jethwa A., Bittner T., Eichenlaub U., Suridjan I., Kollmorgen G. (2023). An accurate fully automated panel of plasma biomarkers for Alzheimer’s disease. Alzheimers Dement..

[189] Tsiknia A.A., Edland S.D., Sundermann E.E., Reas E.T., Brewer J.B., Galasko D., Banks S.J., Alzheimer’s Disease Neuroimaging Initiative (2022). Sex differences in plasma p-tau181 associations with Alzheimer’s disease biomarkers, cognitive decline, and clinical progression. Mol. Psychiatry.

[190] Angioni D., Delrieu J., Hansson O., Fillit H., Aisen P., Cummings J., Sims J.R., Braunstein J.B., Sabbagh M., Bittner T. (2022). Blood Biomarkers from Research Use to Clinical Practice: What Must Be Done? A Report from the EU/US CTAD Task Force. J. Prev. Alzheimers Dis..

[191] Mattke S., Cho S.K., Bittner T., Hlavka J., Hansson M. (2020). Blood-based biomarkers for Alzheimer’s pathology and the diagnostic process for a disease-modifying treatment: Projecting the impact on the cost and wait times. Alzheimers Dement..

[192] Angioni D., Hansson O., Bateman R.J., Rabe C., Toloue M., Braunstein J.B., Agus S., Sims J.R., Bittner T., Carrillo M.C. (2023). Can We Use Blood Biomarkers as Entry Criteria and for Monitoring Drug Treatment Effects in Clinical Trials? A Report from the EU/US CTAD Task Force. J. Prev. Alzheimers Dis..

[193] Kubota M., Bun S., Takahata K., Kurose S., Momota Y., Iwabuchi Y., Tezuka T., Tabuchi H., Seki M., Yamamoto Y. (2025). Plasma biomarkers for early detection of Alzheimer’s disease: A cross-sectional study in a Japanese cohort. Alzheimers Res. Ther..

[194] Burton J., Brothers H.M., Hutchison R.M., Murphy J., Sun T., Dent G., Curiale G., Kowalski K. (2025). Lower baseline amyloid beta burden is associated with greater percent of amyloid beta positron emission tomography reduction and better clinical outcomes in the aducanumab Phase 3 trials ENGAGE and EMERGE in early Alzheimer’s disease. J. Prev. Alzheimers Dis..

[195] Ornago A.M., Pinardi E., Grande G., Valletta M., Calderón-Larrañaga A., Andersson S., Calvani R., Picca A., Marzetti E., Winblad B. (2025). Blood biomarkers of Alzheimer’s disease and 12-year muscle strength trajectories in community-dwelling older adults: A cohort study. Lancet Healthy Longev..

[196] Stevenson-Hoare J., Heslegrave A., Leonenko G., Fathalla D., Bellou E., Luckcuck L., Marshall R., Sims R., Morgan B.P., Hardy J. (2023). Plasma biomarkers and genetics in the diagnosis and prediction of Alzheimer’s disease. Brain.

[197] Tanne J.H. (2025). FDA approves blood test to diagnose Alzheimer’s. BMJ.

[198] Bagger F.O., Borgwardt L., Jespersen A.S., Hansen A.R., Bertelsen B., Kodama M., Nielsen F.C. (2024). Whole genome sequencing in clinical practice. BMC Med. Genom..

[199] Rankin S.M., Marskell L., Hamad L., Machin L. (2025). The UK National screening committee, the newborn genomes programme, and the ethical conundrum for UK newborn screening. J. Community Genet.

